# In vivo non-invasive staining-free visualization of dermal mast cells in healthy, allergy and mastocytosis humans using two-photon fluorescence lifetime imaging

**DOI:** 10.1038/s41598-020-71901-2

**Published:** 2020-09-10

**Authors:** Marius Kröger, Jörg Scheffel, Viktor V. Nikolaev, Evgeny A. Shirshin, Frank Siebenhaar, Johannes Schleusener, Jürgen Lademann, Marcus Maurer, Maxim E. Darvin

**Affiliations:** 1 Department of Dermatology, Venerology and Allergology, Berlin Institute of Health, Charité-Universitätsmedizin Berlin, Corporate Member of Freie Universität Berlin, Humboldt-Universität zu Berlin, Charitéplatz 1, 10117 Berlin, Germany; 2 Faculty of Physics, Tomsk State University, Lenin Ave. 36, 634050 Tomsk, Russia; 3 Faculty of Physics, Lomonosov Moscow State University, Leninskie gory 1/2, 119991 Moscow, Russia

**Keywords:** Biophysical methods, Imaging, Immunological techniques, Microscopy, Imaging the immune system, Innate immune cells

## Abstract

Mast cells (MCs) are multifunctional cells of the immune system and are found in skin and all major tissues of the body. They contribute to the pathology of several diseases including urticaria, psoriasis, atopic dermatitis and mastocytosis where they are increased at lesional sites. Histomorphometric analysis of skin biopsies serves as a routine method for the assessment of MC numbers and their activation status, which comes with major limitations. As of now, non-invasive techniques to study MCs in vivo are not available. Here, we describe a label-free imaging technique to visualize MCs and their activation status in the human papillary dermis in vivo. This technique uses two-photon excited fluorescence lifetime imaging (TPE-FLIM) signatures, which are different for MCs and other dermal components. TPE-FLIM allows for the visualization and quantification of dermal MCs in healthy subjects and patients with skin diseases. Moreover, TPE-FLIM can differentiate between two MC populations in the papillary dermis in vivo—resting and activated MCs with a sensitivity of 0.81 and 0.87 and a specificity of 0.85 and 0.84, respectively. Results obtained on healthy volunteers and allergy and mastocytosis patients indicate the existence of other MC subpopulations within known resting and activated MC populations. The developed method may become an important tool for non-invasive in vivo diagnostics and therapy control in dermatology and immunology, which will help to better understand pathomechanisms involving MC accumulation, activation and degranulation and to characterize the effects of therapies that target MCs.

## Introduction

Mast cells (MCs) derive from hematopoietic progenitor cells. They are not found in the circulation but reside in almost every organ, in particular in tissues which form the barrier between and the environment and the host. They are versatile immune cells with diverse functions in both homeostasis and pathological conditions^1^. Their role in the development of immunoglobulin E–associated acute allergic reactions^2^, contribution to bacterial and fungal infection immunity^3^, tuberculosis^4^, cardiac fibrosis^5^, mastocytosis and related disorders^6–8^, urticaria^9,10^, promotion of Alzheimer’s disease pathogenesis^11^, inflammation^12,13^, arteriogenesis^14^ and the regulation of the epidermal barrier function^15^ is well documented. Being part of the immune system, cutaneous MCs are present in the dermis with increased counts in its superficial layer—the papillary dermis^16^. They are strategically localized and accumulate in “sub-compartments” of the skin in close proximity to nerve fibers^17^ blood and lymphatic vessels and hair follicles^18^, which allows the transmition of local inflammatory signals to distant target cells and tissues^1^. Under physiological conditions, MCs appear to be in a resting state^19^. MCs are metabolically heterogeneous and cytoplasmic granules’ maturation occurs throughout their entire life cycle^20^. Immature MCs contain a few large cytoplasmic granules with a few vesicles and lower histamine content^21^. In the process of maturation, MCs accumulate biologically active substances including heparin, histamine and various proteases including tryptase and chymase in their granules, which decrease in size and increase in number during differentiation^22^. The size of these granules ranges from 0.3 to 2 µm, while the size of dermal MCs is ≈10 µm^23^.

**Figure 1:**
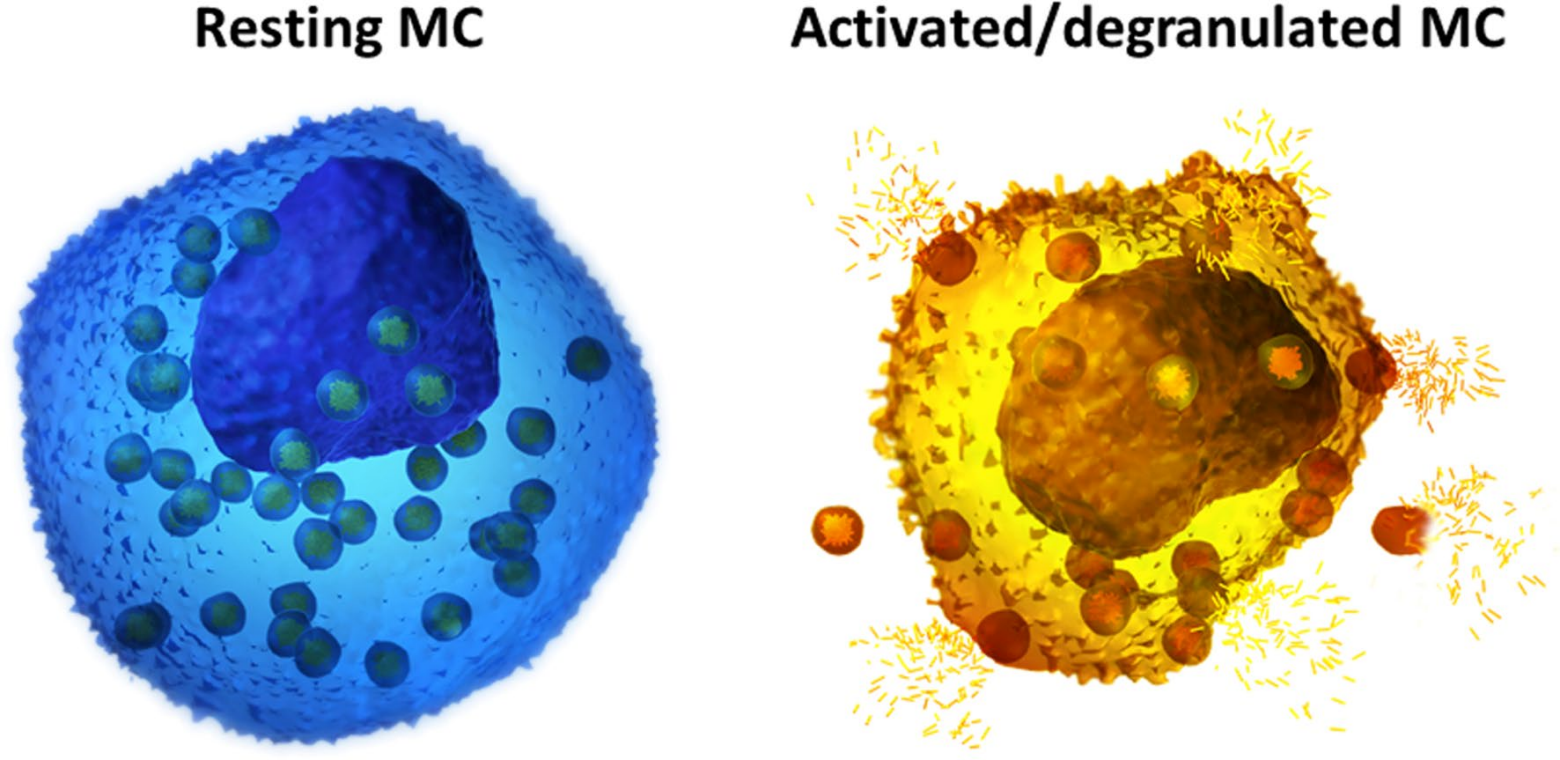
Schematic illustration of resting (left) and activated/degranulated (right) MCs.

MC activation via IgE-dependent and -independent stimuli induce a process of rapid release of their granular content into the extracellular space, a process called degranulation, leading to increasing vascular permeability and orchestration of the inflammatory responses^24^. In addition, lipid mediators such as prostaglandins and leukotrienes as well as cytokines and chemokines are generated by de novo synthesis and then secreted^23,25–27^. Resting and activated/degranulated MCs are illustrated schematically in Fig. 1.

MCs are non-homogeneously distributed in the skin, and their concentration is increased at sites of inflammation in various pathological conditions including urticaria, psoriasis, atopic dermatitis and mastocytosis, where they are held to impact on the pathogenesis^12,28^. However, it is not yet clear how the accumulation of MCs contributes to local immunological reactions since MCs have been shown to exhibit both, inflammatory, as well as anti-inflammatory functions^29,30^. Also, MC accumulation may occur without any immediate pathophysiological consequences^10^.

To date, the routine method for visualization of MCs in the skin is immunohistochemistry of punch biopsies^31^, which requires surgical intervention and cannot be performed in patients with wound healing disorders. Other methods like electron microscopy and atomic force microscopy^1,32^ are also not applicable in vivo. The imaging of fluorescent MCs has been described in the skin of transgenic mice via in vivo two-photon microscopy^18,33^. This technique has conceivable advantages over conventional in vivo microscopic procedures^34^. It allows, for example, for monitoring of fluorescence-labeled cell migration^35,36^. Using this technique, Tong et al*.*^37^ developed the “immune atlas” of the skin, which describes the distribution of immune cells across different locations in the skin. However, in this study, the in vivo experiments were performed on anesthetized transgenic mice using fluorescent labelling.

The intrinsic fluorescence signal of the dermis originates from all dermal components and, therefore, the component-specific fluorescence is superimposed, making the fluorescence-based separation of certain molecules challenging^38,39^. Recently presented multi-channel two-photon/SHG (second harmonic generation)/three-photon/THG (third harmonic generation) in vivo rat tissue imaging^40^ does not separate between cell types. In contrast, in vivo imaging of dermal MCs without labeling or the use of fluorescent tags in murine skin has been reported by Li et al.^41^ based on the tryptophan-originated autofluorescence, which is not MC-specific.

There are clinical situations where a non-invasive technique for visualizing skin MCs may help to better manage patients, e.g. improve diagnosis (urticaria, mastocytosis, mastocytoma) without the need for a biopsy, to monitor the disease course and treatment responses in single cells. Current methods have major limitations such as the requirement for serial skin biopsies which would additionally come from different locations making a direct comparison difficult. Label-free in vivo imaging can overcome these challenges and allow for the investigation of MCs and their responses at a single cell level, at the same location over a long time period.

Thus, in order to solve the challenges associated with in vivo visualization of MCs in human skin, an imaging technique which is able to separate between component-specific fluorescence contributions and to measure at depths exceeding 70 µm, is required. Two-photon tomography in combination with the fluorescence lifetime imaging (TPT/FLIM) perfectly fulfills these requirements. The TPT/FLIM imaging technique allows to observe and screen skin in vivo deeper than 200 µm^42^, and provides the potential to separate between dermal components based on differences in two-photon excited fluorescence lifetime imaging (TPE-FLIM) signatures. This was recently shown for the main extracellular matrix proteins and capillaries of the papillary dermis in vivo^43^.

The aim of this study was to visualize human skin MCs in vivo with a non-invasive and staining-free approach.

## Results

### Characteristic TPE-FLIM signature of primary human skin MCs in vitro

**Figure 2:**
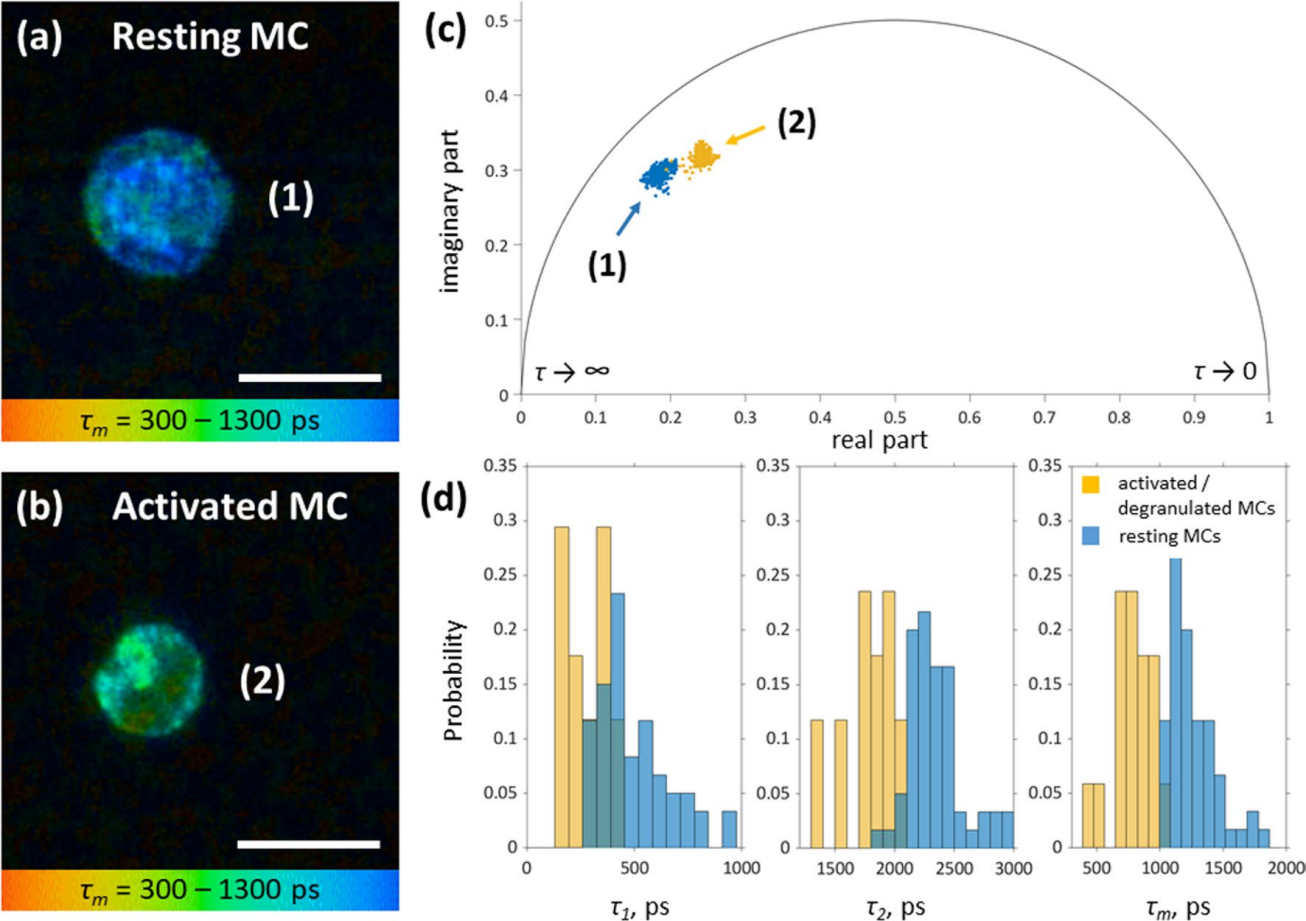
TPE-FLIM parameters of hsMCs in vitro. TPE-FLIM *τ*_*m*_ image (mean fluorescence lifetime *τ*_*m*_ in the 300–1,300 ps range) of hsMCs in vitro: Primary human skin MCs were prepared from skin obtained from breast reduction surgery and cultured for 5 days. **(a)** MCs were washed with PBS and imaged directly or **(b)** were incubated with ionomycin at 1 µM for 15 min before imaging. TPE-FLIM parameters *τ*_*1*_, *τ*_*2*_ and *τ*_*m*_ were recorded with laser excitation at 760 nm with 100 fs pulses and a repetition rate of 80 MHz at 3–5 mW. Scale bar: 10 µm. **(c)** Phasor plot showing distribution of τ_m_ for resting (blue, MC (1)) and ionomycin-treated, activated (orange, MC (2)) hsMCs. **(d)** Distribution of TPE-FLIM parameters *τ*_*1*_, *τ*_*2*_ and *τ*_*m*_ for resting (blue, *n* = 43) and ionomycin-treated, activated (orange, *n* = 13) MCs.

Cultured primary human skin MCs (hsMCs) were analyzed using TPT/FLIM. MCs were characterized by homogenous and densely packed cytoplasmic granules, had a close-to-spherical shape, and were ≈10 µm in size (Fig. 2a). Ionomycin-induced MC activation resulted in the loss of cell membrane adjacent granules and a decrease in MC size by ≈1.5 µm (Fig. 2b) from 10.2 ± 0.8 to 8.7 ± 0.4 µm.

Exteriorized granules and released content were occasionally seen in the culture medium. In addition, β-hexosaminidase activity was detected in the culture supernatant indicating degranulation (data not shown). The appearance of activated/degranulated MCs was less homogenous as seen in the *τ*_*m*_ distribution and less round in shape compared to non-activated resting MCs. Analyses of the TPE-FLIM parameters (*τ*_*1*_, *τ*_*2*_, *τ*_*m*_, *a*_*1*_/*a*_*2*_ and average two-photon excited autofluorescence (TPE-AF) intensity) revealed significant differences (*p* < 0.0001 for all parameters) between both groups with higher TPE-AF intensity and longer *τ*_*m*_ for resting MCs (*n* = 43) compared to lower TPE-AF intensity and shorter *τ*_*m*_ for ionomycin-treated, activated/degranulated (*n* = 13) MCs (Fig. 2a–d; Table 1). 30% of the in vitro observed MCs are degranulated, while the remaining 70% are in a resting state.

**Table 1:** TPE-FLIM parameters *τ*_*1*_, *τ*_*2*_, *τ*_*m*_, *a*_*1*_/*a*_*2*_ and TPE-AF intensity of the pure and IgE-sensitized resting and activated/degranulated MCs, of the main dermal cells measured in vitro in the cell culture and of the shorter and longer *τ*_*m*_ MCs measured in vivo on the volar forearm of healthy volunteers, allergic and mastocytosis patients and ex vivo in human skin cryo-sections.

	Number of cells	*τ*_*m*_ in ps	*τ*_*1*_ in ps	*τ*_*2*_ in ps	*a*_*1*_/*a*_*2*_	TPE-AF intensity, photons/mW
Resting MCs	43	1,248 ± 287	533 ± 266	2,289 ± 317	1.5 ± 0.5	1,300 ± 400
Activated/degranulated MCs	13	862 ± 268	288 ± 130	1920 ± 287	2.5 ± 2.0	900 ± 200
IgE-sensitized resting MCs	14	1,107 ± 54	412 ± 83	2,427 ± 256	1,9 ± 0.3	800 ± 80
IgE-sensitized activated/degranulated MCs	15	747 ± 44	217 ± 21	1877 ± 117	2.1 ± 0.6	1,000 ± 120
Macrophages (short lifetime)	34	461 ± 175	225 ± 84	1,289 ± 278	4.8 ± 3.4	3,000 ± 500
Macrophages (long lifetime)	20	1,348 ± 188	1,016 ± 220	2,432 ± 239	2.6 ± 1.0	800 ± 200
Dendritic cells	14	1,265 ± 180	434 ± 188	2,578 ± 328	1.6 ± 0.2	538 ± 258
Fibroblasts	6	921 ± 81	429 ± 51	1983 ± 137	0.5 ± 0.1	469 ± 137
Neutrophils	21	1,074 ± 109	714 ± 250	1795 ± 600	1.5 ± 0.5	500 ± 115
In vivo (healthy skin) MCs (longer *τ*_*m*_)	71	1,171 ± 117	344 ± 49	2,301 ± 77	1.5 ± 0.3	300 ± 40
In vivo (healthy skin) MCs (shorter *τ*_*m*_)	48	678 ± 151	241 ± 59	1947 ± 230	3.0 ± 1.1	800 ± 100
In vivo (mastocytosis skin) MCs (longer *τ*_*m*_)	9	1,211 ± 130	360 ± 76	2,243 ± 141	1.3 ± 0.2	280 ± 40
In vivo (mastocytosis skin) MCs (shorter *τ*_*m*_)	3	597 ± 136	173 ± 31	1902 ± 206	3.5 ± 0.8	700 ± 100
In vivo (allergy skin) MCs (longer *τ*_*m*_)	8	970 ± 157	310 ± 58	2,204 ± 103	1.8 ± 0.7	320 ± 120
In vivo (allergy skin) MCs (shorter *τ*_*m*_)	9	546 ± 89	185 ± 20	1925 ± 147	4.6 ± 1.1	840 ± 160
Ex vivo (skin biopsy) MCs (longer *τ*_*m*_)	9	1,204 ± 97	417 ± 86	2,181 ± 166	1.7 ± 1.0	1,600 ± 300
Ex vivo (skin biopsy) MCs (shorter *τ*_*m*_)	4	730 ± 140	243 ± 43	1743 ± 167	2.4 ± 0.7	2000 ± 300

In the tissue, MCs are sensitized by IgE bound to the high affinity receptor FcεR1 occupying most receptors. However, over time in culture the amount of MC-bound IgE decreases. To investigate the effect of IgE sensitization on the TPE-FLIM signature, cultured hsMCs were sensitized with human IgE by incubation with 1 µg/ml of purified plasma IgE for 1 h. As shown in the IgE-sensitized MC culture in vitro (Table 1), TPE-FLIM parameters *τ*_*1*_ and *τ*_*m*_ of MCs slightly decreased after sensitization of MCs with IgE (*p* < 0.05) for both resting and activated MC populations.

The distribution of TPE-FLIM parameters *τ*_*1*_, *τ*_*2*_ and *τ*_*m*_ of the major constituents of MC granules heparin and histamine, and corresponding phasor plot are presented in Supplementary Fig. 1. Tryptase has very weak TPE-AF intensity and therefore is not presented. The comparison of the distribution of TPE-FLIM parameters and phasor plots for MCs in vitro (Fig. 2c,d) and heparin and histamine (Supplementary Fig. 1b) reveals an indication of the existence of heparin and histamine in the MCs. However, the decreased TPE-AF intensity of histamine and heparin compared to NAD(P)H minimizes the influence on the FLIM parameters of the MCs.

A detailed analysis of the TPE-FLIM parameters obtained from bi-exponential regression of the fluorescence decay data is presented in the Supplementary Fig. 2. A clear separation is visible for *τ*_*1*_(*τ*_*2*_), *τ*_*1*_(*a*_*1*_/*a*_*2*_), *τ*_*2*_(*a*_*1*_/*a*_*2*_) and *τ*_*2*_((*a*_*1*_− *a*_*2*_)/(*a*_*1*_ + *a*_*2*_)) plots. Hence, resting and activated/degranulated MCs can be readily distinguished by their distinct TPE-FLIM parameters.

### Ex vivo dermal cell specific immunofluorescence confirms in vitro identification of MCs

Comparison of TPE-FLIM parameters of main papillary dermis cells, such as macrophages, dendritic cells and fibroblasts measured in vitro in the cell culture showed that resting and activated/degranulated MCs can be distinguished from other cell types (Table 1, Supplementary Fig. 3), indicating their potential successful visualization in the skin ex vivo and in vivo.

**Figure 3:**
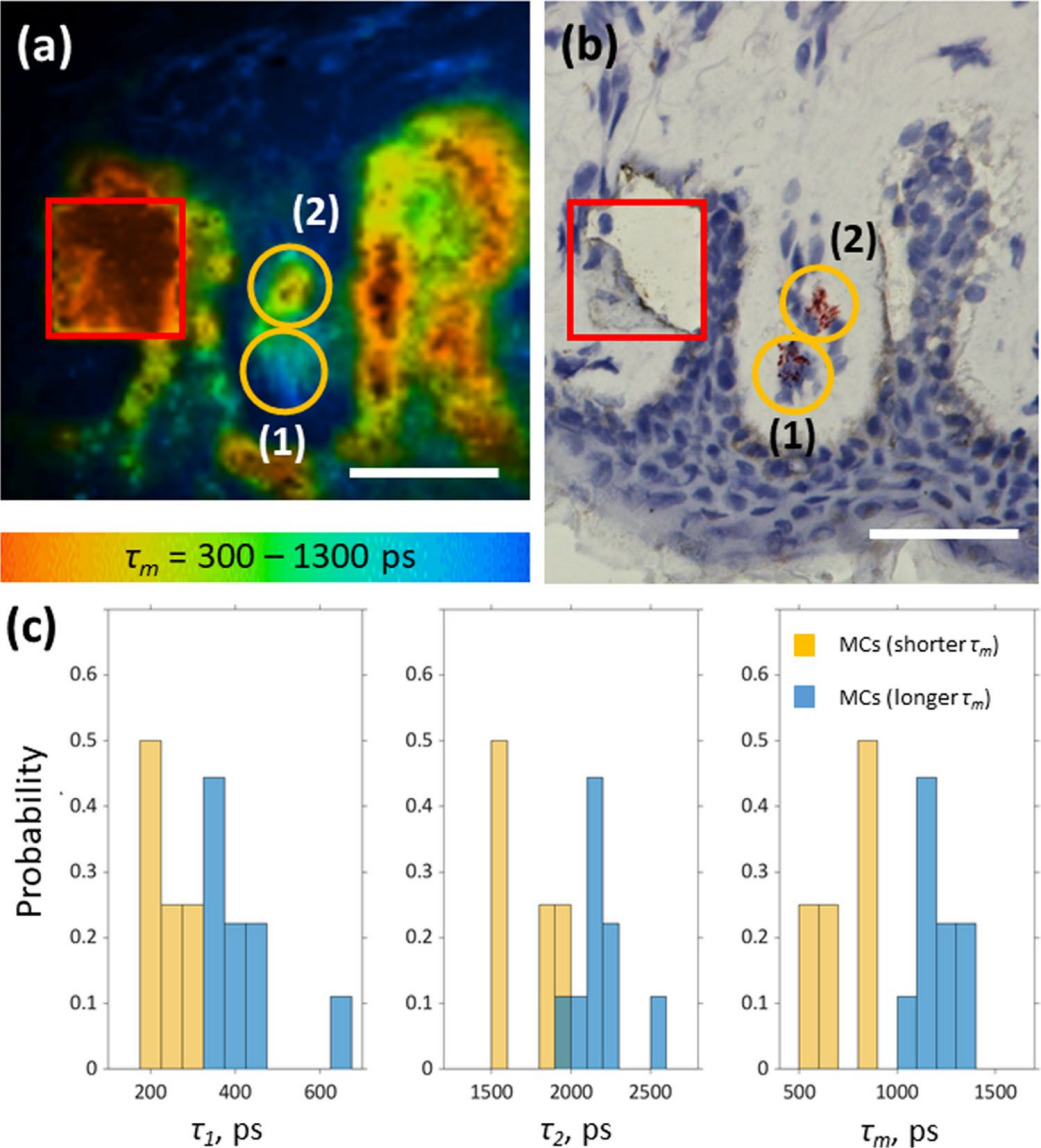
MC ex vivo verification using TPE-FLIM and bright field microscopy. Side by side comparison of TPE-FLIM image (mean fluorescence lifetime *τ*_*m*_ in the 300–1,300 ps range) **(a)** and corresponding brightfield microscopic image after staining of human skin biopsy cryo-sections for MCs with tryptase and counter stained nuclei with Mayer's hemalum solution **(b)** and a histogram of TPE-FLIM parameters measured for the 17 staining-proved MCs (nine MCs with a longer *τ*_*m*_ and eight MCs with a shorter *τ*_*m*_)** (c)**. The MCs ((1) and (2) in **a** and **b**) are marked with orange ellipses and the laser-burned labels (28 µm × 28 µm) are marked in red. Images have been rotated and zoomed to match the same orientation and size. Scale bar: 30 µm.

To further confirm the specificity of the MC signature, side by side comparison of human skin cryo-sections was performed ex vivo, which were first analyzed by TPT/FLIM and then followed by conventional immunohistochemistry staining with tryptase. As marked in orange ellipses in Fig. 3, the suspected MCs (Fig. 3a) are superimposed with the MCs, verified by staining (Fig. 3b). The contrast of shorter lifetimes of MCs compared to the longer lifetimes of the collagen and elastin dense dermis makes MCs distinguishable. In an effort to increase the sensitivity and specificity for MC visualization additional criteria such as MC size and shape were used. Moreover, in the TPE-FLIM signatures of these skin cryo-sections the MCs match the population of the MCs with a longer *τ*_*m*_ (1 in Fig. 3a) and MCs with a shorter *τ*_*m*_ (2 in Fig. 3a), measured in vitro in the culture as resting and activated/degranulated MCs (Table 1). The proportion of observed activated/degranulated MCs is ≈44%. The TPE-FLIM parameters of the staining-proved MCs determined ex vivo are summarized in Table 1. The enlarged TPE-AF signal intensity is potentially related to the dehydration of the skin biopsy samples.

**Figure 4:**
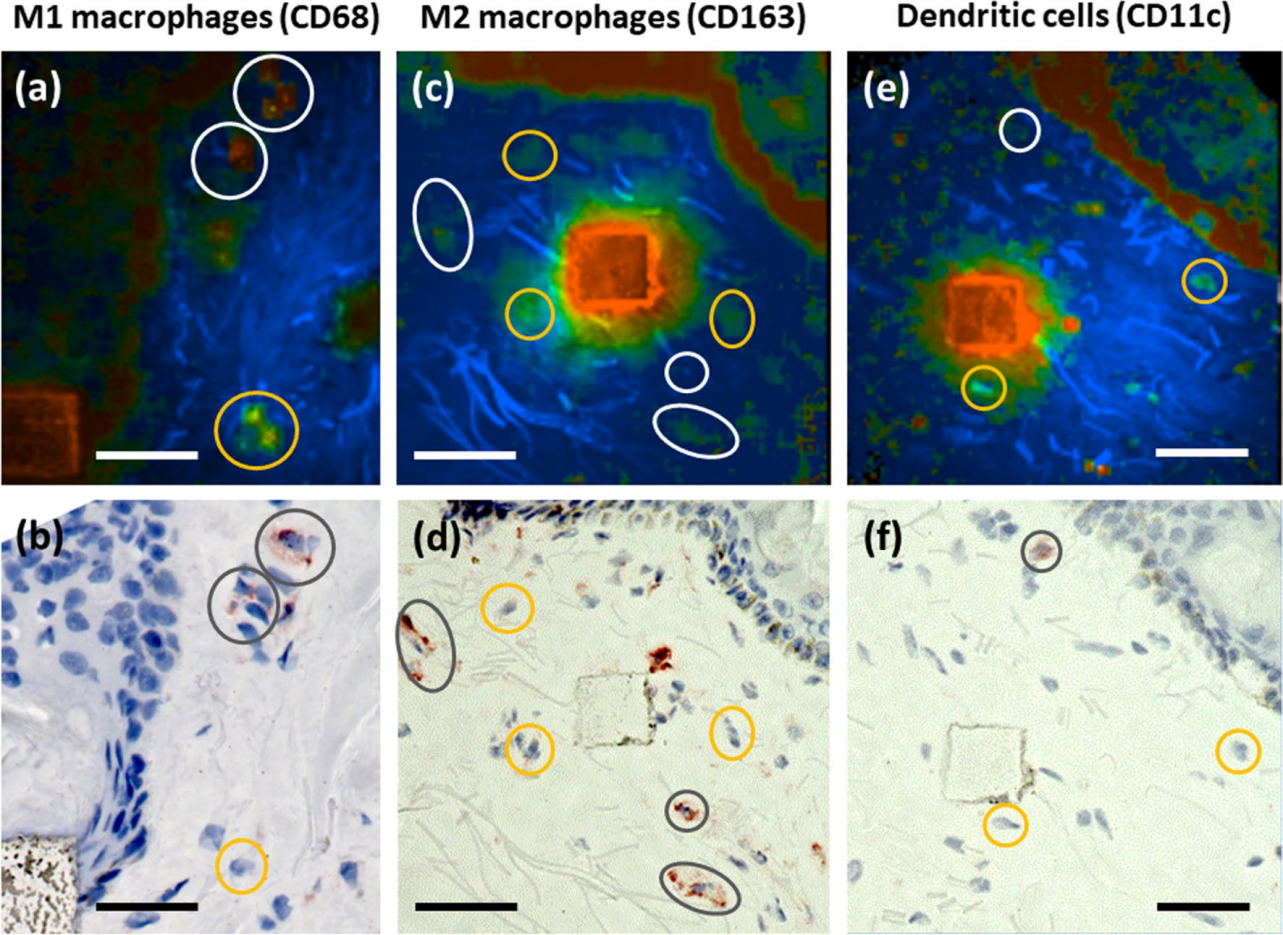
Macrophage and dendritic cell ex vivo verification using TPE-FLIM and bright field microscopy. Side by side comparison of TPE-FLIM images (mean fluorescence lifetime *τ*_*m*_ in the range 300–1,300 ps) **(a, c, e)** and corresponding light microscopic images **(b, d, f)** after staining of human skin biopsy cryo-sections for M1 macrophages with CD68 (b), M2 macrophages with CD163 **(d)** and dendritic cells with CD11c **(f)** and counterstained nuclei with Mayer's hemalum solution. MCs are marked with orange circles, macrophages and dendritic cells with white/gray circles, respectively. The laser-burned labels are 28 µm × 28 µm. Images have been rotated and zoomed to match the same orientation and size. Scale bar: 30 µm.

The 2D correlations of the TPE-FLIM parameters for the staining-proved MCs with a longer *τ*_*m*_ (*n* = 9) and MCs with a shorter *τ*_*m*_ (*n* = 4) measured ex vivo are presented in Supplementary Fig. 4 and show good separation in *τ*_*1*_(*τ*_*2*_), *τ*_*1*_(*a*_*1*_/*a*_*2*_), *τ*_*2*_(*a*_*1*_/*a*_*2*_) and *τ*_*2*_((*a*_*1*_-*a*_*2*_)/(*a*_*1*_ + *a*_*2*_)) plots, which is similar to the separation performed for in vitro measurements (Supplementary Fig. 2). To exclude false positive signals from other immune cells in the papillary dermis, sections were further stained for macrophages and dendritic cells and overlaid with the corresponding TPE-FLIM images (Fig. 4, white/gray circles). Using the developed algorithm, neither macrophages nor dendritic cells were erroneously assigned as MCs (Fig. 4, orange circles).

Fibroblasts, the most concentrated cells of the dermis, have normally an elongated shape and are not visible in the skin in vivo due to their low TPE-AF intensity in comparison to the surrounding extracellular matrix (mainly elastin). However, fibroblasts can be visualized in in vitro collagen matrix (Supplementary Fig. 5a–d). In the cell culture, fibroblasts are round in shape and their TPE-AF intensity is comparable to other dermal cells. However, fibroblasts are spreading in the collagen matrix and adopt their elongated shape. The TPE-FLIM parameters for six fibroblasts, measured in the collagen matrix, are shown in the Supplementary Fig. 5e and are summarized in Table 1.

In summary, all cells that were recorded and marked by TPE-FLIM stained positive for tryptase in immunohistochemistry demonstrating TPE-FLIM signatures to be a reliable tool to identify MCs in frozen skin biopsies.

### TPT/FLIM identifies human skin MCs of healthy volunteers in vivo

**Figure 5:**
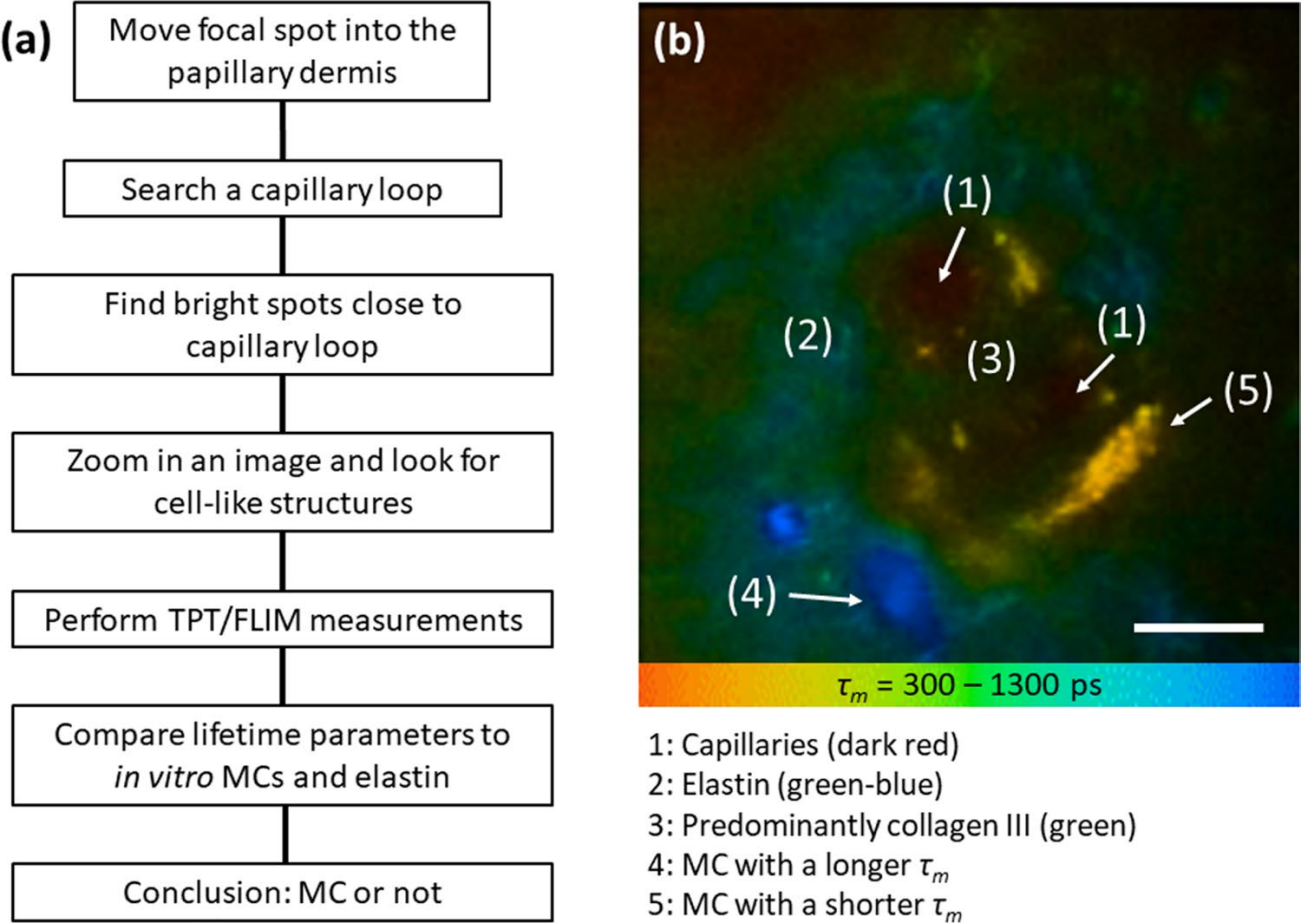
MC in vivo search algorithm using TPE-FLIM method. TPT/FLIM imaging of human dermal MCs in vivo: Flowchart of the search algorithm for the identification of MCs in vivo using TPT/FLIM **(a)** and a typical papillae TPE-FLIM image showing blood capillaries (1) surrounded with elastin (2) and collagen III (3) and located in close proximity to blood capillary MCs characterized with a longer *τ*_*m*_ (> 1,000 ps) (4) and a shorter *τ*_*m*_ (< 800 ps) (5), measured in vivo without staining in the volar forearm at 80 µm depth **(b)**. The mean fluorescence lifetime *τ*_*m*_ is shown by the color gradient ranging from 300 to 1,300 ps. Scale bar: 10 µm.

Based on our in vitro and ex vivo data we hypothesized that MCs’ TPE-FLIM signatures can be clearly identified in the skin in vivo using TPT/FLIM. To identify the MCs, we developed a search algorithm, which is presented as flowchart in Fig. 5a. This algorithm assumes that skin MCs are present in the dermis and thus are expected at depths exceeding 70 µm, directly inside the dermal papillae, in close proximity to the blood capillaries. Thus, the focal spot of TPT should be moved into the papillary dermis and the capillary loop should be found by fast scanning (for instance, 3.2 s per image, typical image size ≈150 µm × 150 µm, power 25–50 mW) regime. The dermal cells have higher TPE-AF intensity than surrounding tissue in the dermis (the epidermal content is usually highly fluorescent). Therefore, “bright fluorescent spots” should be found and zoomed in to find cell-like structures. MCs should have an average size of ≈ 10 µm with close-to-round shape in loose connective tissue and an elongated to ovoid shape when in close proximity to blood vessels^44,45^. When such a cell is found, a TPT/FLIM image should be recorded and the TPE-FLIM parameters should be compared with known TPE-FLIM parameters of resting and activated/degranulated MCs (Fig. 2, Table 1). Matching of these TPE-FLIM parameters indicates if the measured cell is resting or activated/degranulated MC. A representative example of TPE-FLIM visualized MCs in the papillae is presented in Fig. 5b. The image was recorded from the skin of a healthy volunteer at the inner forearm at the depth of 80 µm. Capillaries (1 in Fig. 5b) surrounded with elastin (2 in Fig. 5b) and collagen III (3 in Fig. 5b) and two MC populations characterized by low TPE-AF intensity (≈ 300 photons/mW) and longer *τ*_*m*_ (> 1,000 ps) (4 in Fig. 5b) as well as by high TPE-AF intensity (≈ 800 photons/mW) and a shorter *τ*_*m*_ (< 800 ps) (5 in Fig. 5b) are clearly visible in the TPE-FLIM image of the papilla.

**Figure 6:**
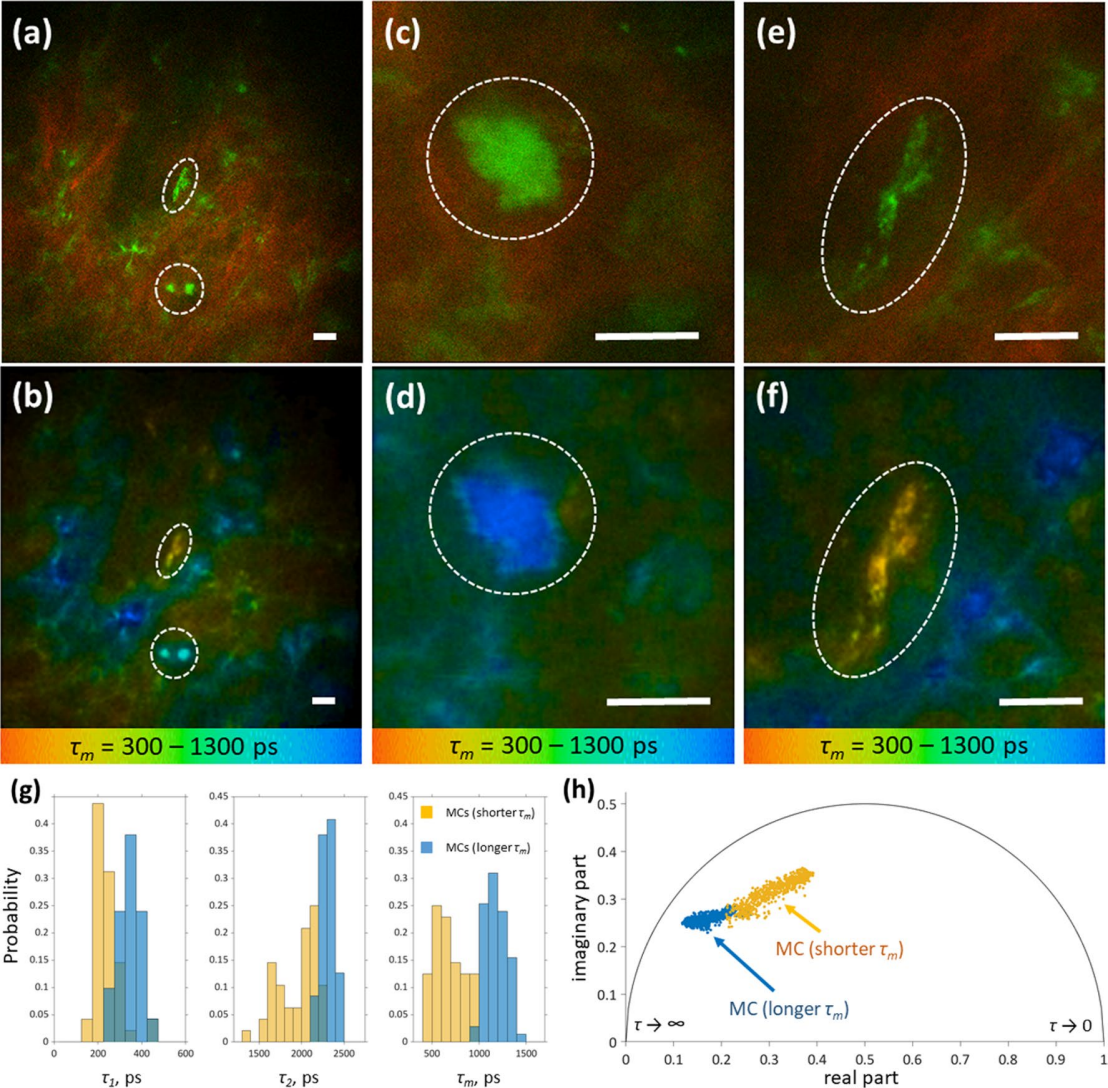
Differentiation of MC activation status in vivo. Top row: merged TPE-AF (green) and SHG (red) images of the human dermis measured in vivo without staining on the inner forearm of a 40 y. o. healthy male volunteer at 90 µm **(a)**, 95 µm **(c)** and 90 µm depth **(e)**. The corresponding TPE-FLIM images (bottom row, **b, d,** **f**), show the mean fluorescence lifetimes *τ*_*m*_ in a color gradient from 300 to 1,300 ps. The marked dotted areas show the MCs **(a, b)**. Zoomed images of MCs with a longer *τ*_*m*_ **(c, d)** and shorter *τ*_*m*_ **(e, f)** are shown. Scale bar: 10 µm. Distribution of TPE-FLIM parameters *τ*_*1*_, *τ*_*2*_ and *τ*_*m*_ for two MC populations, such as MCs with a longer *τ*_*m*_ (*n* = 71, blue) and MCs with a shorter *τ*_*m*_ (*n* = 48, orange), measured in vivo without staining in the inner forearm skin of 28 human volunteers **(g)** and the phasor plot of one exemplary MC with a longer *τ*_*m*_ (blue points) and one MC with a shorter *τ*_*m*_ (orange points) measured in vivo and presented in **(d)** and **(f)** **(h)**.

An exemplary application of this algorithm is demonstrated in Fig. 6. Taking the MC density into consideration, determined as ≈174 ± 120 MCs/mm^2^ for 10 µm biopsies in the papillary dermis using MC-specific staining of cryo-sections (Supplementary Fig. 6), we assume that an image of ≈150 µm × 150 µm will contain ≈1–4 MCs over the whole depth of 120 µm, which was observed in the experiments. In the first stage, during the TPT fast scanning (3.2 s per image, typical image size ≈150 µm × 150 µm), “bright fluorescent spots” of 10 µm and certain shape, established in vitro and ex vivo were searched in the papillary dermis (typical depths ≈80–120 µm) using TPE-AF/SHG imaging (Fig. 6a).

The TPE-AF intensity of the “bright fluorescent spots” was considerably higher in comparison to the fluorescence background of the surrounding extracellular matrix, whose fluorescence intensity is due to elastin and collagen^43,46^ (Fig. 5b). Subsequently, the image magnification was increased in order to focus on the “bright fluorescent spots” (Fig. 6c, e) and the TPE-FLIM images (Fig. 6d, f) were recorded for further analysis. A cell-like structure with the size of inner granules between 0.5 and 1.5 µm, that is typical for MCs^47^, was often visible (Fig. 6d, f). Using this algorithm, we recorded 119 cells from 28 healthy volunteers and compared the TPE-FLIM parameters to those of MCs measured in vitro*.* The TPE-FLIM parameters are summarized in the Table 1.

The recorded dermal cells could be grouped according to their TPE-FLIM parameters into cells with longer *τ*_*m*_ (> 1,000 ps) and shorter *τ*_*m*_ (< 800 ps). The obtained TPE-FLIM parameters (Table 1) match well with the ones obtained from cultured hsMCs before and after activation with ionomycin (Fig. 2c; Table 1) suggesting that the recorded dermal cells are MCs of two populations. However, TPE-AF intensities were inversed. MCs with a longer *τ*_*m*_ presented with a significantly lower intensity compared to cells with a shorter *τ*_*m*_ (Table 1). The reason for this difference remains to be investigated. MCs with a longer *τ*_*m*_ (Fig. 6c, d) had a circular cell body and substructures barely distinguishable, due to tightly packed granules. In contrast, MCs with a shorter *τ*_*m*_ (Fig. 6e, f) had an elongated shape and clearly distinguishable granularly substructure. In total, 119 MCs were recorded. Analysis of the TPE-FLIM signatures revealed 71 MCs with a longer *τ*_*m*_ and 48 MCs with a shorter *τ*_*m*_ (Fig. 6g, h, Table 1).

Importantly, TPE-FLIM signatures of the two MC populations identified in vivo are significantly different (*p* < 0.0001 for *τ*_*1*_, *τ*_*2*_, *τ*_*m*_, *a*_*1*_/*a*_*2*_ and average TPE-AF intensity, Table 1) and resemble these of cultured MCs in vitro (Figs. 1, 6; Table 1). Figure 6h shows the phasor plot of one exemplary MC with a longer *τ*_*m*_ (blue points) and one MC with a shorter *τ*_*m*_ (orange points) measured in vivo and presented in Fig. 6d, f. Both MC populations are partly superimposed in the phasor plot, but can be well separated.

The 2D correlations of TPE-FLIM parameters for MCs with a longer *τ*_*m*_ (*n* = 71) and MCs with a shorter *τ*_*m*_ (*n* = 48) measured in vivo are presented in Supplementary Fig. 7 and show good separation in *τ*_*1*_(*τ*_*2*_), *τ*_*1*_(*a*_*1*_/*a*_*2*_), *τ*_*2*_(*a*_*1*_/*a*_*2*_) and *τ*_*2*_((*a*_*1*_− *a*_*2*_)/(*a*_*1*_ + *a*_*2*_)) plots, which are similar to the separation of resting and activated/degranulated hsMC populations measured in vitro (Supplementary Fig. 2).

Approximately 67% of the dermal MCs corresponds to the MC population with a longer *τ*_*m*_, thus matches the resting MC population from in vitro experiments. However, the MCs with a shorter *τ*_*m*_, matching the activated MC population measured in in vitro experiments, could be also detected in vivo. Their visualization is much easier due to the higher TPE-AF intensity (Table 1).

The presented results for the decision tree classifier were calculated for 407 cells, the cell distribution is described in the materials and methods section*.* The parameters for the classifier are criteria of quality of a split is Gini impurity, minimal samples for split is 2, maximum depth of the tree is 6, and the samples had equal weight. The test set was 40% of the whole set and the training set included the remaining 60% of the whole set and split was tested 1,000 times. The first model classified MCs against other cells. The sensitivity was 0.86 ± 0.03 and the specificity was 0.82 ± 0.07, which suggests a high accuracy of 0.84 for the TPE-FLIM method for MCs. When activated/degranulated MCs were classified the sensitivity was 0.87 ± 0.04 and the specificity 0.84 ± 0.04. For resting MCs, the sensitivity was 0.81 ± 0.06 and the specificity was 0.85 ± 0.03 (Supplementary Fig. 8). These results meet our expectations for a robust classification. Thus, we see that resting and activated/degranulated MCs can be separated from other cells in the skin with high accuracy without additional staining.

### TPT/FLIM identifies human skin MCs of mastocytosis and allergy patients in vivo

**Figure 7:**
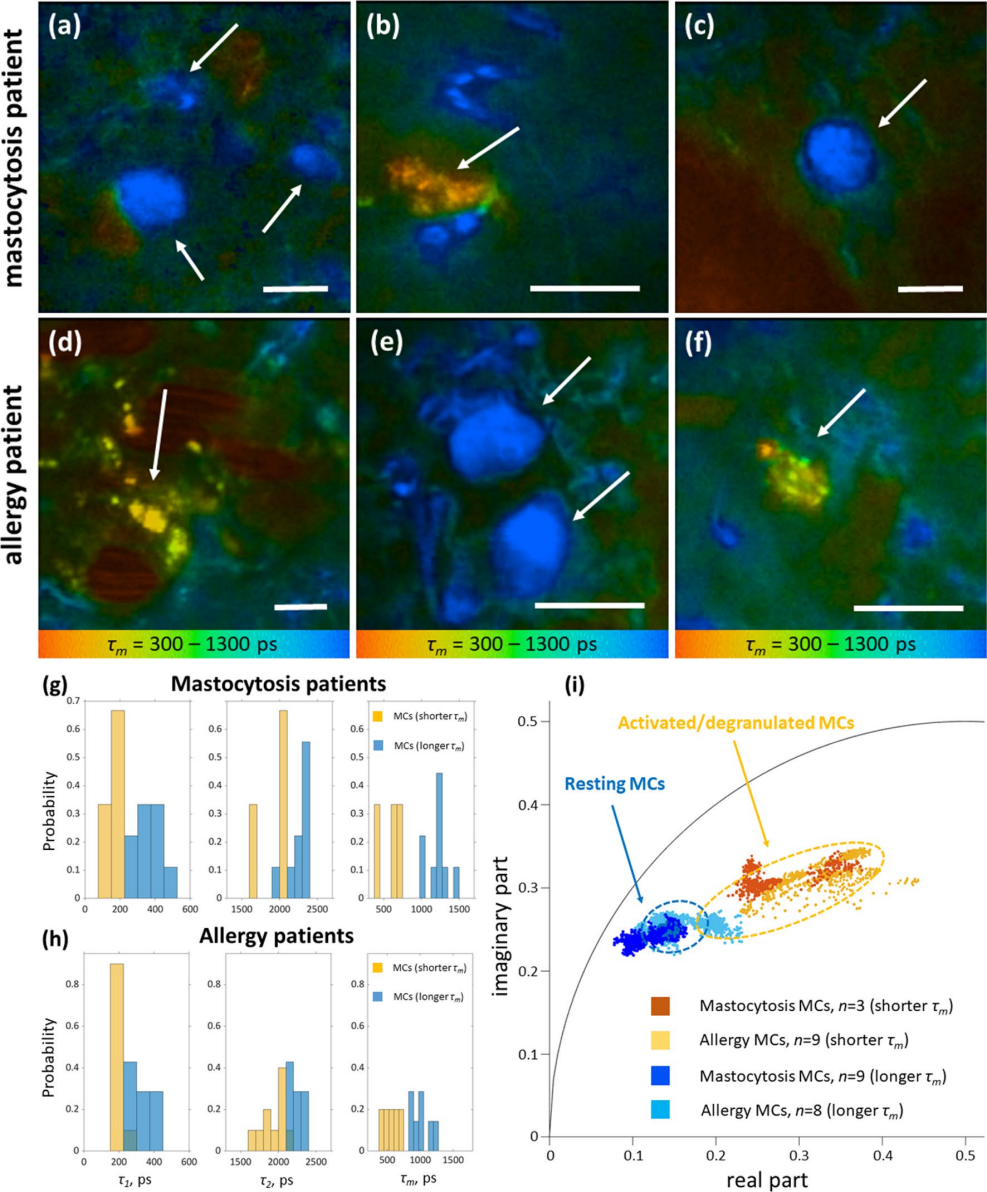
MC TPE-FLIM imaging of mastocytosis and allergy patients. TPE-FLIM typical MC images of human dermis in vivo, showing mean fluorescence lifetime *τ*_*m*_ in color gradient from 300 to 1,300 ps, in the disease-affected forearm area of the mastocytosis patient **(a–c)** and in the asymptomatic forearm area of the allergy patient **(d–f)**. MCs are shown with arrows. The images were recorded at 760 nm excitation wavelength, 50 mW laser power and 6.8 s acquisition time in the depth of 90 µm. Scale bar: 10 µm. Distribution of TPE-FLIM parameters *τ*_*1*_, *τ*_*2*_ and *τ*_*m*_ for MCs in the skin of mastocytosis patients (*n* = 12) **(g)** and in the skin of allergy patients (*n* = 17) **(h)** and the phasor plot of the resting MCs with a longer *τ*_*m*_ (dark blue points—mastocytosis, *n* = 9; light blue points—allergy, *n* = 8) and activated/degranulated MCs with a shorter *τ*_*m*_ (dark orange points—mastocytosis, *n* = 3; light orange points—allergy, *n* = 9). The marked dotted areas show the distribution for resting (*n* = 6, blue) and activated/degranulated MCs (*n* = 6, orange) measured in vivo in healthy volunteers.

TPE-FLIM images from four mastocytosis patients (total of 12 cells) and three allergy patients (total of 17 cells) were further recorded. For one allergy patient the IgE in serum is increased at 110.0 kU/l compared to the reference value of < 85.0 kU/l and increased IL-8 at 314 pg/ml compared to < 15 pg/ml, which can be produced by MCs^48^. In the lesional skin of mastocytosis patients, the number of MCs was visually higher as compared to non-lesional skin areas and the skin of healthy volunteers. As in volunteers, the majority of MCs were of the longer *τ*_*m*_ population (resting MCs). Importantly, the appearance of MCs in the lesional and non-lesional dermis of mastocytosis patients was similar to that of MCs of healthy volunteers (see Figs. 6c, 5d). Dermal mastocytosis MCs were round or slightly elongated in shape and ≈10 µm in size with very densely packed granules (Fig. 7a–c). Also, TPE-FLIM signatures of MCs recorded from mastocytosis patients with longer *τ*_*m*_ (*n* = 9) as well as shorter *τ*_*m*_ (*n* = 3) were not significantly different from MCs of healthy volunteers (Table 1; Fig. 7a–c), showing ≈ 75% resting MCs in mastocytosis and ≈ 60% resting MCs in healthy subjects. In contrast, a majority of MCs recorded from the allergy patient were MCs with a shorter *τ*_*m*_ (Table 1; Fig. 7d–f). The cytoplasmic granules of 0.5–1.5 µm size showed a high TPE-AF intensity, which was not previously observed. Moreover, this patient had visually more MCs of both populations compared to healthy volunteers.

## Discussion

In this study, we demonstrate for the first time, that the non-invasive TPT/FLIM technique can identify MCs in the papillary dermis of healthy and diseased human skin in vivo and distinguish them from other cell types and dermal structures by specific TPE-FLIM signatures. In addition, in vivo TPE-FLIM imaging can differentiate between two skin MC populations, characterized by either longer or shorter *τ*_*m*_, which match the TPE-FLIM signatures of resting and activated cultured MCs, respectively. Ionomycin-induced MC activation in vitro results in significant (*p* < 0.0001) changes in all TPE-FLIM parameters (*τ*_*1*_, *τ*_*2*_, *τ*_*m*_) towards shorter lifetimes (Fig. 2d). Importantly, ionomycin itself did not show any detectable fluorescence signal. Therefore, changes in TPE-FLIM parameters are the result of cellular changes. The phasor plot, showing the fluorescence decay parameters’ distribution for two representative MCs, indicated in Fig. 2a, b, that blue points correspond to the longer *τ*_*m*_ (1 in Fig. 2a) and orange points to the shorter *τ*_*m*_ (2 in Fig. 2b) MC phenotypes, which can be distinguished in vitro and in vivo (Figs. 2c, Fig. 6). The TPE-FLIM parameters are characteristic for MCs and distinguishes them from other cells in the papillary dermis, such as macrophages, dendritic cells, neutrophils and fibroblasts (Table 1, Fig. 4, Supplementary Fig. 3). Therefore, the TPE-FLIM signature can be used to visualize MCs in human skin in vivo.

Considering the presence of different cell types in the dermis, the identification of the origin of the fluorescence signal is crucial to distinguish between different cell types. Generally, at 760 nm excitation, the fluorescence signal from cells is due to the presence of free and bound NAD(P)H^49^ with fluorescence lifetime components centered at *τ*_*1*_≈3 60 ps and *τ*_*2*_≈ 2,200 ps^50,51^. For different cell types, as well as for changes in cell metabolism or activation redistribution between NAD(P)H forms and its binding to different enzymes may cause pronounced changes in TPE-FLIM signatures, which enables the separation between cells at different stages of differentiation, normal and cancer cells, cells with oxidative stress, etc.^39,51–54^. In fact, it is possible that NAD(P)H fluorescence decay parameters could be characteristic for different cell types. For MCs, in addition to the NAD(P)H, content of MC granules could contribute to the TPE-FLIM signatures. In in vitro experiments, activation and degranulation i.e. the release of granular content from MCs, resulted in a marked ≈ 1.5-fold decrease in fluorescence intensity at 760 nm excitation, supporting of the role of granules in the formation of a MC-related fluorescence signal. MC granules contain various preformed mediators including high (up to 1 M^55^) concentrations of histamine, heparin, tryptase as well as serotonin. Although being non-fluorescent by excitation in the visible spectrum, solutions of histamine and heparin exhibited pronounced fluorescence emission at 760 nm two-photon excitation (Supplementary Fig. 1), probably due to the formation of oxidation products or aggregation-induced fluorescence^56–58^. Moreover, the comparison of the phasor plots for MCs in vitro (Fig. 2c) and heparin and histamine solutions (Supplementary Fig. 1b) reveals a correlation between heparin, histamine and the MC activation state. The TPE-FLIM distributions presented in Supplementary Fig. 1 are significantly different from the parameters of NAD(P)H^50,51^. Moreover, the data indicate an impact of IgE molecules bound to the high affinity receptor FcεRI on the TPE-FLIM signature, in particular *τ*_*1*_ and *τ*_*m*_ (Table 1). These data lets suggest that TPE-FLIM signatures might be MC-specific, yet further investigations are required to reveal the exact nature of fluorescence in the MCs and the FLIM signal.

For in vivo applications focusing on MCs, it is essential to selectively and specifically differentiate dermal MCs from other TPE-FLIM signals in the dermis, originating from the extracellular matrix, whose fluorescence is mainly provided by elastin and collagen III. The data show that dermal MCs have a higher TPE-AF intensity and are visible as “bright fluorescent spots” of specific shape, size and *τ*_*m*_ distribution in the skin (Fig. 6a). Importantly, their TPE-FLIM parameters are significantly different to those of the surrounding extracellular matrix components, such as elastin, collagen III and blood capillaries (green–blue and dark red in the FLIM images, Fig. 6b, Supplementary Fig. 3) and match the TPE-FLIM parameters of cultured MCs measured in vitro (Fig. 2d, Table 1). Moreover, a precursor for the presence of cells in the dermis is the collagen I-free niches, which are visible by measuring the collagen I-specific SHG signal (red in Fig. 6a, c, e). In addition, MCs can be clearly distinguished from other dermal cells such as fibroblasts, dendritic cells and macrophages. They are localized in the proximity of blood capillaries, which are visible in dark red in the TPE-FLIM images (Fig. 6b, d, f) due to ultrafast fluorescence decay of Hb photoproducts^59^. The presence of cytoplasmic granules is a specific hallmark feature of MCs. Additionally, the size of measured cells was always ≈ 10 µm, which is specific for MCs. As can be seen in the phasor plot (Supplementary Fig. 3), TPE-FLIM parameters of resting MCs and fibroblasts are partly superimposed. However, fibroblasts are characterized with substantially lower TPE-AF intensity in comparison to MCs. Moreover, the size of fibroblasts in the dermis (> 20 µm) is much larger than of other dermal cells (MCs, macrophages, dendritic cells, etc.) with a typical size of ≈10 µm, and taking into account that fibroblasts do not contain distinct granules, it can be concluded that fibroblasts cannot be visualized in the dermis in vivo at the detection parameters used in this work. Therefore, fibroblasts could not be mistaken as MCs in the skin during in vivo or ex vivo experiments. It should be taken into consideration that due to lower TPE-AF intensity of resting MCs, their concentration in the skin could be underrepresented and, thus their percentage is most likely higher than the reported 67%.

The ex vivo human skin biopsies stained for the presence of MCs were measured using TPT/FLIM (Fig. 3). The ex vivo MCs with a longer *τ*_*m*_ shows an elongated shape that had never been observed in the in vivo experiments. In total, 17 histology-matched MCs (nine MCs with a longer *τ*_*m*_ and eight MCs with a shorter *τ*_*m*_) were recorded, which all showed an elongated shape and a size below 10 µm, which is probably a result of stress induced during freezing, excision, water loss and staining of the skin biopsies in the ex vivo experiment, which can initiate spontaneous MC degranulation and changes of MC size, shape and elasticity in comparison to the in vivo experiment. As can be seen in Fig. 3a, the orange-marked areas have different *τ*_*m*_ values than the main extracellular matrix component elastin (*τ*_*m*_ = 1,600 ± 110 ps). The nucleus is not visible, probably due to the masking of the TPE-AF by the granules’ signals, which are observed in this image as clouded or dotted points, as confirmed in the staining image (Fig. 3b). The images do not show a perfect superposition. Yet, the burned square and the epidermal features ensure the same skin position. The distortion is most likely caused by the staining of the biopsy section.

Under physiological in vivo conditions, human dermal MCs should be in a resting state and their degranulation is induced by IgE-independent triggers such as neurotransmitters or complement or IgE-mediated, e.g. by cross-linking of surface-bound IgE with corresponding antigen/allergen^2,60,61^. MC activation induces changes in the TPE-FLIM signal and a shift towards a shorter *τ*_*m*_. However, degranulation could not be confirmed in ex vivo and in vivo experiments. Therefore, it cannot be definitely concluded, that the measured MCs with a shorter *τ*_*m*_ correspond to degranulated MCs. They could also correspond to MCs in an early differentiation stage, or even to a distinct MC population with a distinct composition of MC granules. For this reason, MCs with a shorter *τ*_*m*_ are called activated/degranulated MCs. These aspects should be a subject of future studies.

In the allergy patient with increased IgE value the fluorescence lifetimes are shorter, similar to the in vitro results, which is an additional indicator that the observed dermal cells are indeed MCs. The TPE-FLIM parameters of MCs in allergy and healthy skin were only slightly different (*p* > 0.05) with obvious differences presented in the phasor plot for the resting MCs (Fig. 7i). MCs of allergy patients with a longer *τ*_*m*_ determined as resting MCs show a broader distribution with a minor superposition with the activated/degranulated MC population of healthy volunteers. The broader distribution of the resting MCs in allergy compared to healthy indicate that resting MCs are presented in at least two subpopulations in allergic skin. The reason for that can be an influence of allergy on the MC maturation or degranulation processes, which require additional investigations on a larger cohort of patients. In the mastocytosis patients, a higher density of MCs than in healthy subjects was observed. This leads to the conclusion that the observed MC distribution is consistent with the criteria for mastocytosis with a higher percentage of resting MCs compared to allergy and normal skin. The TPE-FLIM parameters of MCs in mastocytosis and healthy skin were only slightly different (*p* > 0.05). However, some differences are obvious in phasor plot for both resting and activated/degranulated MC populations (Fig. 7i). It is obvious that each MC population consists of at least two subpopulations. This assumption as well as the origin of the observed changes should be a topic of future investigations. It is unknown that there are significant differences in IgE in mastocytosis patients compared to healthy volunteers. It is assumed that the resting and activated/degranulated MC populations are identical between healthy and diseased skin, however the results of this pilot study performed on allergy and mastocytosis patients suggest an existence of other MC populations, which should be proved in future investigations.

Data for different types of cells can be partially divided based on the TPE-FLIM parameters and the phasor approach, however, there are superimposed regions that do not allow 100% separation. Since various parameters were determined, such as shape of the cells, TPE-AF intensity and bi-exponential approximation of lifetime decay, and representation of data in the phase space, it was decided to combine some of these data and construct a classification rule to search for hidden dependencies. The basic decision tree classifier, which is available in the public domain and was proven in practice, was used^62^. The feature vector was created by combining the characteristics described in the materials and methods section and had the final dimension: 1 (circular form) + 1 (normalized TPE-AF intensity of cell normalized by area and power) + 256 (decay curve) + 7 (TPE-FLIM parameters obtained after bi-exponential approximation of decay curve) = 265. The exponential model is described in the statistical analysis and classification algorithm section. The MC size in vivo could be slightly different from MCs measured in vitro in suspension and ex vivo in biopsies, therefore the MC size was not included in the feature vector for the classification model. As a classification accuracy, two parameters, sensitivity and specificity were chosen. These parameters reflect the quality of the classification and indicate Type I and Type II errors. The classification algorithm is robust with the decision tree classifier showing a sensitivity of 0.87 ± 0.04 and a specificity 0.84 ± 0.04 for activated/degranulated MCs. For resting MCs, the sensitivity was 0.81 ± 0.06 and the specificity was 0.85 ± 0.03. For the classification of MCs of both populations versus other cells, the sensitivity is 0.86 ± 0.03 and the specificity is 0.82 ± 0.07. The diagnostic ability is shown as a ROC-curve in Supplementary Fig. 8. Despite the relatively low number of entries, for a machine learning approach, future challenges are to integrate the algorithm into a pipeline and to optimize the training data set in order to further increase the sensitivity and specificity. Considering these results, it is concluded that the observed “bright fluorescent spots” (Fig. 6) matching presented specific criteria are dermal MCs, which are characterized either by the longer *τ*_*m*_ (Fig. 6d) or shorter *τ*_*m*_ (Fig. 6f). Both MC populations can be well distinguished from each other (Fig. 6), other dermal cells and extracellular matrix components in the papillary dermis (Supplementary Fig. 3). In order to show time course results of the same MC before and after activation treatment (such as ionomycin or IgE) the MC should be stabilized during the in vitro measurement, or activation should be performed during the ex vivo measurement, which is currently not realized and serves a promising future task.

The presented results demonstrate the possibility to identify, track and characterize MCs in human skin in vivo in a non-invasive and staining-free mode using the TPT/FLIM technique. To the best of our knowledge, selective in vivo staining-free imaging of dermal MCs and potentially also other cell types in human skin has, as of yet, not been described. Hence, our data let suggest a possible extension of the TPT/FLIM technique for the assessment of MC number and function within the dermal composition in vivo.

## Materials and methods

### Two-photon tomography in combination with fluorescence lifetime imaging (TPT/FLIM)

A two-photon tomograph (TPT, Dermainspect, JenLab GmbH, Jena, Germany) equipped with a tunable femtosecond Ti:sapphire laser (Mai Tai XF, Spectra Physics, USA) operated at 760 nm excitation and 100 fs pulses at a repetition rate of 80 MHz, was used for horizontal imaging of cultured human skin MCs (hsMCs) and skin biopsy cryo-sections at 3–5 mW and human skin papillary dermis in vivo at 40–50 mW. The low excitation power of 3–5 mW was applied in order to exclude potential photochemical processes or laser influence on cells. For 760 nm excitation, a 410–680 nm band pass filter was used to detect two-photon excited autofluorescence (TPE-AF) and a 375–385 nm band pass filter was used to detect the second harmonic generation (SHG) signal, which is specific to collagen I. The acquisition time to record one image was 6.8 s. The lateral and axial resolutions were approx. 0.5 µm and 1.2–2.0 µm, respectively ^63^. Two-photon excited fluorescence lifetime imaging (TPE-FLIM) data were processed using the SPCImage software version 8.0 (Becker&Hickl, Berlin, Germany). Fluorescence lifetime decay in each pixel was determined by bi-exponential non-linear regression using the weighted least squares method, i.e. with fast (*τ*_*1*_, *a*_*1*_) and slow (*τ*_*2*_, *a*_*2*_) lifetime and amplitude decay components. The temporal shift of the signal in relation to the instrument response function was fixed. The intensity threshold was chosen depending on the image quality. The fluorescence lifetime decay was binned over the 48 neighboring pixels (binning value 3), yielding photon count values larger than 200 photons/mW for in vivo measurements and larger than 800 photons/mW for in vitro and ex vivo measurements, resulting in reduced residual errors. Introducing the average fluorescence intensity of the cell as the photon count at a binning value of 3, the intensity of the pixel in focus and the 48 neighboring pixels were calculated. The obtained lifetimes (*τ*_*1*_ and *τ*_*2*_) and amplitudes (*a*_*1*_ and *a*_*2*_) were exported and used for the evaluation of the TPE-FLIM distributions and 2D segmentation^43^. The mean lifetime was defined as *τ*_*m*_ = (*a*_*1*_*τ*_*1*_ + *a*_*2*_*τ*_*2*_)/(*a*_*1*_ + *a*_*2*_) and used as a characteristic signature of the fluorescence lifetime decay. The dependences of TPE-FLIM parameters such as *τ*_*1*_(*τ*_*2*_), *τ*_*1*_(*a*_*1*_/*a*_*2*_), *τ*_*2*_(*a*_*1*_/*a*_*2*_) and *τ*_*2*_((*a*_*1*_− *a*_*2*_)/(*a*_*1*_ + *a*_*2*_)) were used for 2D segmentation and separation between different cell types. The *τ*_*1*_/*τ*_*2*_ ratio was calculated by the SPCImage software for each pixel. The TPE-FLIM data were also analyzed and represented in phasor plots, which are based on the transformation of the fluorescence decay data in the frequency domain, whereas the decay is described as amplitude and phase values of the first Fourier component^64^. The phasor plots’ *x*-axis is described by the cosine of the phase value multiplied by the amplitude, the *y*-axis represents the sine of the phase value multiplied with the amplitude^39,65^. The position of the mean lifetime is on the secant from *τ*_*1*_ and *τ*_*2*_, the distance to the circle is given by the proportion of *a*_*1*_ and *a*_*2*_. The TPE-FLIM data were normalized to maximum intensity and the threshold of 70% was set to represent in the phasor plot. The utilized TPT/FLIM system has been previously described in detail elsewhere^43,66^.

### Constituents of MC granules in vitro

Heparin (Biochrom, Berlin, Germany), histamine (Sigma-Aldrich, St. Louis, MO, USA) and tryptase (Sigma-Aldrich, St. Louis, MO, USA) were stored 2 h at room temperature before measurements. TPE-FLIM parameters *τ*_*1*_, *τ*_*2*_ and *τ*_*m*_ were recorded with laser excitation at 760 nm with 100 fs pulses and a repetition rate of 80 MHz at 8 mW.

### Human skin MC cultures

Cultured primary human skin MCs (hsMCs) were prepared as described before^67^. In brief, human breast skin from plastic reduction surgeries was digested in 2.4 U/ml dispase type II (Roche, Basel, Switzerland) over night at 4 °C. The epidermis was removed, the skin was minced with scissors and further digested in PBS containing Ca^2+^ and Mg^2+^ (Gibco, Carlsbad, CA, USA) supplemented with 1% Pen/Strep, 5% FCS, 2.5 µg/ml amphothericin (Biochtrom, Berlin, Germany), 5 mM MgSO_4_, 10 µg/ml DNaseI (Roche, Basel, Switzerland), 0.75 mg/ml H-3506 hyaluronidase (Sigma-Aldrich, St. Louis, MO, USA) and 1.5 mg/ml collagenase type II (Worthington Biochemical Corp., Lakewood, NJ, USA) at 37 °C in a waterbath with shaking for 1 h. The cell suspension was filtered via 300 µm and 40 µm stainless steel sieves (Retsch, Haan, Germany) followed by centrifugation at 300 *g* for 15 min at 4 °C. The digestion cycle was repeated once. Cells were washed in PBS w/o Ca^2+^ and Mg^2+^ (Gibco, Carlsbad, CA, USA), MCs isolated by CD117 positive MACS enrichment (Miltenyi, Bergisch Gladbach, Germany) and cultured in basal Iscove’s medium supplemented with 1% Pen/Strep, 10% FCS, 1% non-essential aminoacids (all Gibco, Carlsbad, CA, USA), 226 µM α-monothioglycerol. After 24 h recombinant human IL-4 (20 ng/ml) and hSCF (100 ng/ml) (both Peprotech, Rocky Hill, NJ, USA) were added for long term cultures. Cells were cultured at 1.0 × 10^6^ cells/ml with addition of IL-4 and SCF twice a week. Purity of MC cultures were routinely checked by flow cytometry for CD117/FcεRI positive cells and was found to be > 95%. For imaging, cells were used after 5 and 15 days in culture, washed twice with PBS and seeded on 18 mm diameter microscope cover glass (VWR, Darmstadt, Germany) in PBS containing Ca^2+^ at room temperature. IgE sensitization was performed by incubation with human IgE (MerckMillipore, Burlington, Ma, USA) purified from myeloma plasma for 1 h at 37 °C in the culture medium. For degranulation experiments, MCs were incubated with the ionophore Ionomycin (Sigma Aldrich, St. Louis, MO, USA) at 1 µM for 15 min at 37 °C before TPE-FLIM imaging.

### Human dermal fibroblasts in vitro

Human dermal fibroblasts were isolated from the foreskin of a 5 y. o. male as described before^68^. The tissue was washed 3 times in PBS (Gibco, Carlsbad, CA, USA) and digested on the dermal side in 2.8 ml/60 cm^2^ dispase type II (Roche, Basel, Switzerland) and incubated for 20 h at 4 °C. The epidermis and dermis was separated using a forceps, the dermal tissue was placed in a tissue culture plate and 1.5 ml FGM (Gibco, Carlsbad, CA, USA) was added. The enzymatic reaction was stopped by Trypsin (all Sigma-Aldrich, St. Louis, MO, USA) with 10 ml FGM consisting of DMEM medium with 10% FCS, 1% glutamine and 1% Pen/Strep (all Gibco, Carlsbad, CA, USA). The cells’ suspension was filtered using a cell strainer and washed with 5 ml PBS. Cells were centrifuged at 130 g for 5 min at 25 °C, the supernatant discarded and suspended in 10 ml PBS. The medium was changed every other day and cultured for 4 days at 37 °C. The purity was regularly controlled optically and reached approx. 90%. The cells were suspended in a collagen matrix NMSC model on the 18 mm diameter microscope cover glass (VWR, Darmstadt, Germany). The fibroblasts were cultured for 26 h at 37 °C before the TPE-FLIM measurements.

### Human dermal macrophages in vitro

Cultured Periocular human dermal macrophages were prepared as described before^69^. In brief, human periocular skin was digested in 2.4 U/ml dispase type II (Roche, Basel, Switzerland) over night at 4 °C. The epidermis was removed, the skin minced with scissors and further digested in PBS containing Ca^2+^ and Mg^2+^ (Gibco, Carlsbad, CA, USA) supplemented with 1% Pen/Strep, 5% FCS, 2.5 µg/ml amphothericin (Biochrom, Berlin, Germany), 5 mM MgSO_4_, 10 µg/ml DNaseI (Roche, Basel, Switzerland), 0.75 mg/ml H-3506 hyaluronidase (Sigma-Aldrich, St. Louis, MO, USA) and 1.5 mg/ml collagenase type II (Worthington Biochemical Corp., Lakewood, NJ, USA) at 37 °C in a waterbath with shaking for 1 h. The cell suspension was filtered via 300 µm and 40 µm stainless steel sieves (Retsch, Haan, Germany) followed by centrifugation at 300 *g* for 15 min at 4 °C. The digestion cycle was repeated once. Cells were washed in PBS w/o Ca^2+^ and Mg^2+^ (Gibco, Carlsbad, CA, USA), Macrophages isolated by Pan Monocyte Isolation Kit (Miltenyi, Bergisch Gladbach Germany) and cultured in basal Iscove’s medium supplemented with 1% Pen/Strep, 10% FCS, 1% non-essential aminoacids, 226 µM α-monothioglycerol (all Gibco, Carlsbad, CA, USA). After 24 h recombinant human IL-4 (20 ng/ml) and hSCF (100 ng/ml) (both Peprotech, Rocky Hill, NJ, USA) were added for long term cultures. Cells were cultured at 5.0 × 10^5^ cells/ml with addition of IL-4 and SCF twice a week. Purity of macrophage cultures were routinely checked to be > 85%^70^. For imaging, cells were used after 3 days in culture, washed twice with PBS and seeded on 18 mm diameter microscope cover glass for imaging (VWR, Darmstadt, Germany) in PBS containing Ca^2+^ (Gibco, Carlsbad, CA, USA) at room temperature.

### Human dendritic cells in vitro

Dendritic cells were differentiated from CD14 positive peripheral blood mononuclear cells by washing in PBS (Gibco, Carlsbad, CA, USA) and ficoll density gradient centrifugation at 350 *g* for 10 min twice and adding 5 ml RPMI medium supplemented with 1% Pen/Strep and 1% FCS (Biochtrom, Berlin, Germany). Cells in the cell suspension are subsequently counted using 20 µl Tryptan Blue (Sigma-Aldrich, St. Louis, MO, USA) in a hemocytometer. Seeding of 2.0 × 10^6^ cells/ml and incubating at 37 °C under 5% CO_2_ was performed for 2 h. The supernatant and non-attached cells were discarded. 500 µl basal Iscove’s medium was added to the cells supplemented with 1% Pen/Strep, 1% glutamin, 5% HSA, (all Gibco, Carlsbad, CA, USA) 100 ng/mL IL-4, 100 ng/mL GM-CSF (both Peprotech, Rocky Hill, NJ, USA) with medium change every other day for 6 days at 37 °C before TPE-FLIM imaging. The purity was regularly controlled and reached approx. 90%.

### Preparation and cryo-sectioning of human skin tissue for combined TPE-FLIM and histomorphometric analysis

To prove that the in vivo measured dermal cells are MCs, nine human skin biopsy cryo-sections were prepared and measured using the TPT/FLIM technique to acquire TPE-FLIM signature of the suspected MCs. The skin biopsies were obtained from the abdominal region of three female patients (31, 40 and 44 y. o., skin type II according to Fitzpatrick classification^71^) after abdominal reduction surgery. Punch biopsies of 6 mm diameter were obtained, snap frozen and stored at − 80 °C before cryo-sectioning. Vertical histological cryo-sections of 10 µm thickness were prepared on a cryostat (Microm Cryo-Star HM 560, MICROM International GmbH, Walldorf, Germany) after embedding in a cryomedium (Tissue Freezing Medium, Leica Biosystems Richmond Inc., Richmond, IL, USA) and placed on 18 mm diameter microscope cover glasses (VWR, Darmstadt, Germany). The anatomical condition of the biopsies was continuously examined using a transmission microscope (Olympus IX 50, Olympus K.K., Shinjuku, Tokyo, Japan). Only high-quality histological sections were used for further analysis.

Cryo-sections were scanned using TPT/FLIM for cells with MC-specific TPE-FLIM signatures and the corresponding TPE-FLIM signature was recorded. To retrieve the TPE-FLIM measured position for histomorphometric analysis, skin biopsies were labeled by burning a squared area of 28 µm × 28 µm located near the suspected MCs. The burning was done with the laser at 760 nm using a power of 50 mW for 3 s at maximal zoom. The TPE-FLIM images containing suspected MCs with the quadratic label were recorded and the same sections were then used for histomorphometric analysis. All incubations were performed at room temperature unless otherwise stated. In brief, sections were fixed for 10 min in cold acetone (− 20 °C) and rinsed in TBS (Agilent, Santa Clara, CA, USA). Sections were blocked with serum-free protein followed by incubation for 1 h with anti-tryptase antibody (clone AA1) diluted 1:1,000 in antibody diluent (all Agilent, Santa Clara, CA, USA). Slides were rinsed three times with TBS and endogenous peroxidase was blocked with 3% H_2_O_2_ in TBS for 5 min followed by incubation with anti-mouse EnVision + labelled polymer (Agilent Technologies, Santa Clara, USA) for 30 min. Slides were rinsed in TBS as before and incubated with AEC substrate-chromogen (Agilent Technologies, Santa Clara, USA) for 10 min. Nuclei were counterstained with Mayer's hemalum solution (Merck, Darmstadt, Germany). Stained MCs have a brown–red color, which enables to visually distinguish them from other cells and the extracellular matrix. After the staining procedure, target MCs and squared labels of the skin sections were identified by light microscopy and overlaid with TPE-FLIM images matching an appropriate magnification and image orientations.

To prove that the TPE-FLIM parameters of other dermal cells, namely, macrophages and dendritic cells do not match or superimpose with parameters of MCs, negative control measurements were performed. The procedure was similar as described for the verification of MCs in the skin biopsies using specific immunofluorescence, but six human skin cryo-sections were stained for the presence of macrophages and two for dendritic cells, respectively.

For staining of macrophages, the macrophage-specific anti-CD68 (clone ab955) (Abcam, Cambridge, UK) and anti-CD163 antibodies (clone GHI/61.1) (Miltenyi, Bergisch Gladbach, Germany) were used to account for M1 and M2 macrophage phenotypes, respectively. For staining of dendritic cells anti-CD11c antibody (clone B-Ly6) (BD Biosciences, Franklin Lakes, NJ, USA) was used after fixing the cryo-section for 10 min in cold acetone (− 20 °C) and rinsed in TBS.

### Label free in vivo imaging

The volar forearm skin of 27 healthy volunteers (25–65 year old, skin type II–III according to Fitzpatrick classification^71^) without declared immune or skin diseases, disease-affected and non-affected skin areas of four untreated mastocytosis patients (40–60 year old, skin type II) and an unsymtomatic forearm of female atopic dermatitis patient (32 year old, skin type II) at thighs and forearms with reported allergies to grass pollen, dogs and cats with immune suppression therapy (oral application of daily 100 mg of Azathioprine, 20 mg cetirizine and 150 mg Ranitidine) were included for non-invasive in vivo screening of the papillary dermis (60–150 µm depths) using the TPT/FLIM technique. The volunteers were screened between April 2018 and November 2019. The oil objective of the TPT/FLIM was covered by a 150 µm thin cover glass slide embedded into a metal ring and fixed to the volunteer’s forearm. A 10 µl droplet of water was placed between the skin surface and cover glass for maintaining the optical contact. The hair was occasionally removed using scissors without influence on the skin integrity. The TPT/FLIM technique does not have any known side effects, considering a correct power-depth profile for the laser.

### Statistical analysis and classification algorithm

TPE-FLIM data for all MCs were recorded, and descriptive statistics was applied using Matlab R2016a (MathWorks, Natick, MA, USA). All values are given as mean ± standard deviation. Differences between distributions were compared using the non-parametric Kolmogorov–Smirnov test and *p* < 0.05 was considered significant. All machine learning classifications were carried out in Python 3.7 utilizing Scikit-learn 0.22 using a Decision tree classifier. The model was trained using training set sizes of 60% of the complete data set, involving all relevant cells and repeated for 1,000 times with randomized training and test sets in every cycle. Results are exported in form of a confusion matrix and subsequent parameters true positive rate (meaning of sensitivity) and true negative rate (meaning of specificity) are presented as mean ± standard deviation. These parameters reflect the quality of the classification and indicate Type I and Type II errors. For the decision tree^72^ the TPE-FLIM parameter variables *τ*_*1*_, *τ*_*2*_, *τ*_*m,*_* a*_*1*_, *a*_*2*_, *a*_*1*_/*a*_*2*_, (*a*_*1*_* − a*_*2*_)/(*a*_*1*_ + *a*_*2*_), TPE-AF intensity, shape (where 1 is circular and 0 is non-circular) and decay curve, were used for each cell measured in vitro, ex vivo and in vivo. The exponential approximation of lifetimes was averaged over the whole cell and the fluorescent intensity normalized per mW power was averaged over 49 pixel. A total of 407 cells, where 85 were MCs in vitro, 17 MCs ex vivo, 148 MCs in vivo, 54 macrophages in vitro, 4 macrophages ex vivo, 58 macrophages in vivo, 14 dendritic cells in vitro, 6 fibroblasts in vitro and 21 neutrophils in vitro were used as input for the model. Given data vectors from ${x}_{i}\in {R}^{n}$, *i* = 1,…,l and a label vector ${y}_{i}\in {R}^{l}$, where a decision tree recursively separates the data into classes. With the mode $m$ represented as $Q$. For each node a split $\theta =(j,{t}_{m})$ has to decide with the feature $j$ and threshold ${t}_{m}$. The node splits the data into subsets ${Q}_{left}(\theta )$ and ${Q}_{right}(\theta )$

$${Q}_{left}(\theta )=(x,y)|{x}_{j}<={t}_{m}$$

$${Q}_{right}(\theta )=Q\backslash {Q}_{right}(\theta )$$

At the mode $m$ the impurity is calculated by the impurity function $H()$

$$G(Q,\theta )={n}_{left}/{N}_{m} H({Q}_{left}(\theta ))+{n}_{right}/{N}_{m} H({Q}_{right}(\theta ))$$

With the parameter for minimised impurities the subsets are recourse until ${N}_{m}=1.$

The classification criterion has values of 0 for activated MCS, 1 for resting MCs and 2 for other cells, 0 for MCs and 1 for other cells for node *m* in the region ${R}_{m}$ and ${N}_{m}$ observation, the proportion of class *k* observations in node $m$ is ${p}_{mk}=1/{N}_{m}\sum_{{x}_{i}\in {R}_{m}}I({y}_{i}=k)$.

The diagnostic ability is presented in form of a receiver operating characteristic (ROC)-curve where the true positive rate is plotted against the false positive rate of the respective outcomes (Supplementary Fig. 8).

### Ethics

Volunteers for intravital microscopy provided their written informed consent before participation. Skin samples taken from plastic skin reduction surgery for MC preparation and all human skin investigated in this study were used after written informed consent was obtained, as approved by the ethics committee of the Charité-Universitätsmedizin Berlin (EA1-078-18, EA4-193-18, EA1-141-12) according to the Declaration of Helsinki (59th WMA General Assembly, Seoul, October 2008).

## Supplementary information

Supplementary Information.

## References

[1] Tikoo S (2018). Imaging of mast cells. Immunol. Rev..

[2] Galli SJ, Tsai M (2012). IgE and mast cells in allergic disease. Nat. Med..

[3] Piliponsky AM, Romani L (2019). The contribution of mast cells to bacterial and fungal infection immunity. Immunol. Rev..

[4] Garcia-Rodriguez KM, Goenka A, Alonso-Rasgado MT, Hernández-Pando R, Bulfone-Paus S (2017). The role of mast cells in tuberculosis: Orchestrating innate immune crosstalk?. Front. Immunol..

[5] Levick SP, Widiapradja A (2018). Mast cells: Key contributors to cardiac fibrosis. Int. J. Mol. Sci..

[6] Komi DEA, Rambasek T, Wöhrl S (2018). Mastocytosis: From a molecular point of view. Clin. Rev. Allergy Immunol..

[7] Molderings GJ, Brettner S, Homann J, Afrin LB (2011). Mast cell activation disease: A concise practical guide for diagnostic workup and therapeutic options. J. Hematol. Oncol..

[8] Theoharides TC, Valent P, Akin C (2015). Mast cells, mastocytosis, and related disorders. N. Engl. J. Med..

[9] Church MK, Kolkhir P, Metz M, Maurer M (2018). The role and relevance of mast cells in urticaria. Immunol. Rev..

[10] Siebenhaar F, Redegeld FA, Bischoff SC, Gibbs BF, Maurer M (2018). Mast cells as drivers of disease and therapeutic targets. Trends Immunol..

[11] Kempuraj D (2017). Mast cell activation in brain injury, stress, and post-traumatic stress disorder and Alzheimer’s disease pathogenesis. Front. Neurosci..

[12] Bonnekoh H, Scheffel J, Kambe N, Krause K (2018). The role of mast cells in autoinflammation. Immunol. Rev..

[13] Pastwińska J, Agier J, Dastych J, Brzezińska-Błaszczyk E (2017). Mast cells as the strength of the inflammatory process. Polish J. Pathol..

[14] Ribatti D (2018). A new role of mast cells in arteriogenesis. Microvasc. Res..

[15] Sehra S, Serezani APM, Ocaña JA, Travers JB, Kaplan MH (2016). Mast cells regulate epidermal barrier function and the development of allergic skin inflammation. J. Invest. Dermatol..

[16] Weber A, Knop J, Maurer M (2003). Pattern analysis of human cutaneous mast cell populations by total body surface mapping. Br. J. Dermatol..

[17] Morellini N, Finch PM, Goebel A, Drummond PD (2018). Dermal nerve fibre and mast cell density, and proximity of mast cells to nerve fibres in the skin of patients with complex regional pain syndrome. Pain.

[18] Obeidy P, Tong PL, Weninger W (2018). Research techniques made simple: Two-photon intravital imaging of the skin. J. Invest. Dermatol..

[19] Metcalfe, D. D. ASH 50th anniversary review mast cells and mastocytosis. *Heal.* (*San Fr.*) **112**, 946–956 (2008). 10.1182/blood-2007-11-078097 PMC2515131 18684881

[20] Combs, J. W. Maturation of rat mast cells an electron microscope study. *J. Cell Biol.* 563–575 (1966). 10.1083/jcb.31.3.563 PMC2107060 5971648

[21] Galli SJ (1982). Mast cell clones : A model for the analysis of cellular maturation effect of sodium butyrate on mast cells synthesis of sulfated glycosaminoglycans. J. Cell Biol..

[22] Combs JW, Lagunoff D, Benditt EP (1965). Differentiation and proliferation of embryonic mast cells of the rat. J. Cell Biol..

[23] Wernersson S, Pejler G (2014). Mast cell secretory granules: Armed for battle. Nat. Rev. Immunol..

[24] Nguyen M, Pace AJ, Koller BH (2014). Age-induced reprogramming of mast cell degranulation. J. Immunol..

[25] Atiakshin D, Buchwalow I, Samoilova V, Tiemann M (2018). Tryptase as a polyfunctional component of mast cells. Histochem. Cell Biol..

[26] Gordon JR, Burd PR, Galli SJ (1990). Mast cells as a source of multifunctional cytokines. Immunol. Today.

[27] Mukai K, Tsai M, Saito H, Galli SJ (2018). Mast cells as sources of cytokines, chemokines, and growth factors. Immunol. Rev..

[28] Kritas SK (2013). Impact of mast cells on the skin. Int. J. Immunopathol. Pharmacol..

[29] Galli, S. J., Grimbaldeston, M. & Tsai, M. Immunomodulatory mast cells: Negative, as well as positive, regulators of immunity. *Nat. Rev. Immunol.* **8** (2008). 10.1038/nri2327 PMC2855166 18483499

[30] Marichal T, Tsai M, Galli SJ (2013). Mast cells: Potential positive and negative roles in tumor biology. Cancer Immunol. Res..

[31] Shukla, S. A., Veerappan, R., Whittimore, J. S., Ellen Miller, L. & Youngberg, G. A. Mast cell ultrastructure and staining in tissue. *Methods Mol. Biol.* **315**, 63–76 (2006). 10.1385/1-59259-967-2:063 16110149

[32] Reber LL, Marichal T, Galli SJ (2015). New models for analyzing mast cell functions in vivo. Laurent..

[33] Schmerse F (2014). In vivo visualization of uterine mast cells by two-photon microscopy. Reproduction.

[34] Germain, R. N., Robey, E. A. & Cahalan, M. D. A Decade of imaging cellular motility and interaction dynamics in the immune system. *Science (80).* **336**, 1676–1681 (2012). 10.1126/science.1221063 PMC3405774 22745423

[35] Kabashima K, Egawa G (2014). Intravital multiphoton imaging of cutaneous immune responses. J. Invest. Dermatol..

[36] Dijkgraaf, F. E. *et al.* Tissue patrol by resident memory CD8+ T cells in human skin. *Nat. Immunol.* **20** (2019). 10.1038/s41590-019-0404-3 31110315

[37] Tong PL (2015). Leukocyte subsets by multiphoton microscopy. J. Invest. Dermatol..

[38] Drozdowicz-Tomsia K (2014). Multiphoton fluorescence lifetime imaging microscopy reveals free-to-bound NADH ratio changes associated with metabolic inhibition. J. Biomed. Opt..

[39] Shirshin EA (2019). Label-free multiphoton microscopy: The origin of fluorophores and capabilities for analyzing biochemical processes. Biochemistry.

[40] You S (2018). Intravital imaging by simultaneous label-free autofluorescence-multiharmonic microscopy. Nat. Commun..

[41] Li C, Pastila RK, Lin CP (2015). Label-free imaging immune cells and collagen in atherosclerosis with two-photon and second harmonic generation microscopy. J. Innov. Opt. Health Sci..

[42] Sdobnov A, Darvin ME, Lademann J, Tuchin V (2017). A comparative study of ex vivo skin optical clearing using two-photon microscopy. J. Biophoton..

[43] Shirshin EA (2017). Two-photon autofluorescence lifetime imaging of human skin papillary dermis in vivo: Assessment of blood capillaries and structural proteins localization. Sci. Rep..

[44] Sandes AF, Medeiros RSS, Rizzatti EG (2015). Diagnosis and treatment of mast cell disorders: Practical recommendations. Sao Paulo Med. J..

[45] Yong LCJ (1997). The mast cell: Origin, morphology, distribution, and function. Exp. Toxicol. Pathol..

[46] König K (2008). Clinical multiphoton tomography. J. Biophoton..

[47] Azouz NP, Hammel I, Sagi-Eisenberg R (2014). Characterization of mast cell secretory granules and their cell biology. DNA Cell Biol..

[48] Möller, A. *et al.* Human mast cells produce IL-8. *J. Immunol.* **151**, 3261 LP–3266 (1993). 8376778

[49] Huang S, Heikal AA, Webb WW (2002). Two-photon fluorescence spectroscopy and microscopy of NAD(P)H and flavoprotein. Biophys. J..

[50] Skala MC (2007). In vivo multiphoton microscopy of NADH and FAD redox states, fluorescence lifetimes, and cellular morphology in precancerous epithelia. Proc. Natl. Acad. Sci..

[51] Blacker TS (2014). Separating NADH and NADPH fluorescence in live cells and tissues using FLIM. Nat. Commun..

[52] Stringari C (2015). In vivo single-cell detection of metabolic oscillations in stem cells. Cell Rep..

[53] Varone A (2014). Endogenous two-photon fluorescence imaging elucidates metabolic changes related to enhanced glycolysis and glutamine consumption in precancerous epithelial tissues. Cancer Res..

[54] Matsui T (2017). Non-labeling multiphoton excitation microscopy as a novel diagnostic tool for discriminating normal tissue and colorectal cancer lesions. Sci. Rep..

[55] Helander HF, Bloom GD (1974). Quantitative analysis of mast cell structure. J. Microsc..

[56] Tikhonova TN (2018). Dissection of the deep-blue autofluorescence changes accompanying amyloid fibrillation. Arch. Biochem. Biophys..

[57] Pansieri J (2019). Ultraviolet-visible-near-infrared optical properties of amyloid fibrils shed light on amyloidogenesis. Nat. Photon..

[58] Semenov, A. N. *et al.* The oxidation-induced autofluorescence hypothesis: Red edge excitation and implications for metabolic imaging. *Molecules* **25** (2020). 10.3390/molecules25081863 PMC7221974 32316642

[59] Shirshin EA (2018). Formation of hemoglobin photoproduct is responsible for two-photon and single photon-excited fluorescence of red blood cells. Laser Phys. Lett..

[60] Cheng LE, Hartmann K, Roers A, Krummel MF, Locksley RM (2013). Perivascular mast cells dynamically probe cutaneous blood vessels to capture immunoglobulin E. Immunity.

[61] Frandsen, P. M., Kortekaas Krohn, I. J. M., Hoffmann, H. J. & Schiøtz, P. O. The influence of IgE on cultured human mast cells. *Allergy Asthma Immunol. Res.* **5**, 409–414 (2013). 10.4168/aair.2013.5.6.409 PMC3810549 24179689

[62] Magana-Mora A, Bajic VB (2017). OmniGA: Optimized omnivariate decision trees for generalizable classification models. Sci. Rep..

[63] Breunig HG (2013). Clinical coherent anti-Stokes Raman scattering and multiphoton tomography of human skin with a femtosecond laser and photonic crystal fiber. Laser Phys. Lett..

[64] Digman MA, Caiolfa VR, Zamai M, Gratton E (2008). The phasor approach to fluorescence lifetime imaging analysis. Biophys. J..

[65] Lakner, P. H., Monaghan, M. G., Möller, Y., Olayioye, M. A. & Schenke-Layland, K. Applying phasor approach analysis of multiphoton FLIM measurements to probe the metabolic activity of three-dimensional in vitro cell culture models. *Sci. Rep.* **7** (2017). 10.1038/srep42730 PMC5304149 28211922

[66] Zhu Y (2014). Penetration of silver nanoparticles into porcine skin ex vivo using fluorescence lifetime imaging microscopy, Raman microscopy, and surface-enhanced Raman scattering microscopy. J. Biomed. Opt..

[67] Guhl S, Artuc M, Neou A, Babina M, Zuberbier T (2011). Long-term cultured human skin mast cells are suitable for pharmacological studies of anti-allergic drugs due to high responsiveness to FcεRI cross-linking. Biosci. Biotechnol. Biochem..

[68] Rittié, L. & Fisher, G. J. Isolation and culture of skin fibroblasts. in *Fibrosis Research: Methods and Protocols* (eds. Varga, J., Brenner, D. A. & Phan, S. H.) 83–98 (Humana Press, 2005). 10.1385/1-59259-940-0:083. 10.1385/1-59259-940-0:083 16118447

[69] Botting RA (2017). Phenotypic and functional consequences of different isolation protocols on skin mononuclear phagocytes. J. Leukoc. Biol..

[70] Nielsen MC, Andersen MN, Møller HJ (2020). Monocyte isolation techniques significantly impact the phenotype of both isolated monocytes and derived macrophages in vitro. Immunology.

[71] Fitzpatrick TB (1988). The validity and practicality of sun-reactive skin types I through VI. Arch. Dermatol..

[72] Breiman, L., Friedman, J. H., Olshen, R. A. & Stone, C. J. *Classification and regression trees*. (Wadsworth & Brooks/Cole Advanced Books & Software, 1984).

