# Supplementary material for: In vivo non-invasive staining-free visualization of dermal mast cells in healthy, allergy and mastocytosis humans using two-photon fluorescence lifetime imaging
Source: Sci Rep. 2020 Sep 10;10:14930. doi: 10.1038/s41598-020-71901-2 (PMC7484787; doi:10.1038/s41598-020-71901-2)
Supplement: Supplementary file 1 — Supplementary Information. [file 41598_2020_71901_MOESM1_ESM.docx]

Supplementary Materials:

***In vivo* non-invasive staining-free visualization of dermal mast cells in healthy, allergy and mastocytosis humans using two-photon fluorescence lifetime imaging**

Marius Kröger^1^, Jörg Scheffel^1^, Viktor V. Nikolaev^1,2^, Evgeny A. Shirshin^3^, Frank Siebenhaar^1^, Johannes Schleusener^1^, Jürgen Lademann^1^, Marcus Maurer^1^, Maxim E. Darvin^1^*

* Corresponding author: E-mail: maxim.darvin@charite.de

^1^ Charité – Universitätsmedizin Berlin, corporate member of Freie Universität Berlin, Humboldt-Universität zu Berlin, and Berlin Institute of Health, Department of Dermatology, Venerology and Allergology, Charitéplatz 1, 10117 Berlin, Germany

^2^ Tomsk State University, Faculty of Physics, 634050, Lenin Ave. 36, Tomsk, Russia

^3^ Lomonosov Moscow State University, Faculty of Physics, 119991, Leninskie gory 1/2, Moscow, Russia


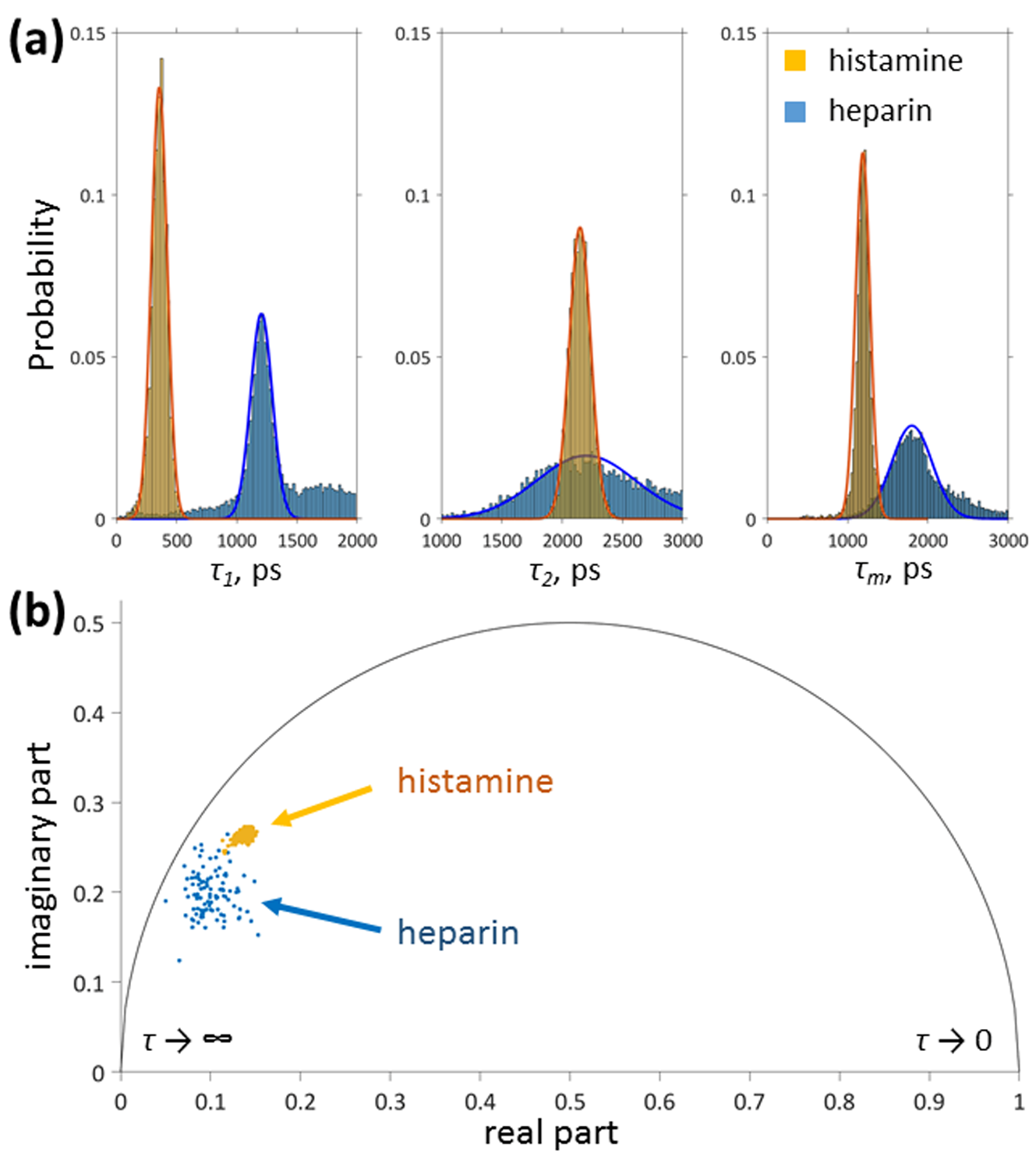


**Supplementary Fig. 1. Differentiation of histamine and heparin.** Distribution of TPE-FLIM parameters *τ_1_*, *τ_2_*, *τ_m_* for histamine (orange) and heparin (blue) (a) and corresponding phasor plot (b). Tryptase is characterized by a very weak fluorescence intensity and therefore is not presented. TPE-FLIM parameters *τ_1_*, *τ_2_* and *τ_m_* were recorded with laser excitation at 760 nm with 100 fs pulses and a repetition rate of 80 MHz at 8 mW.


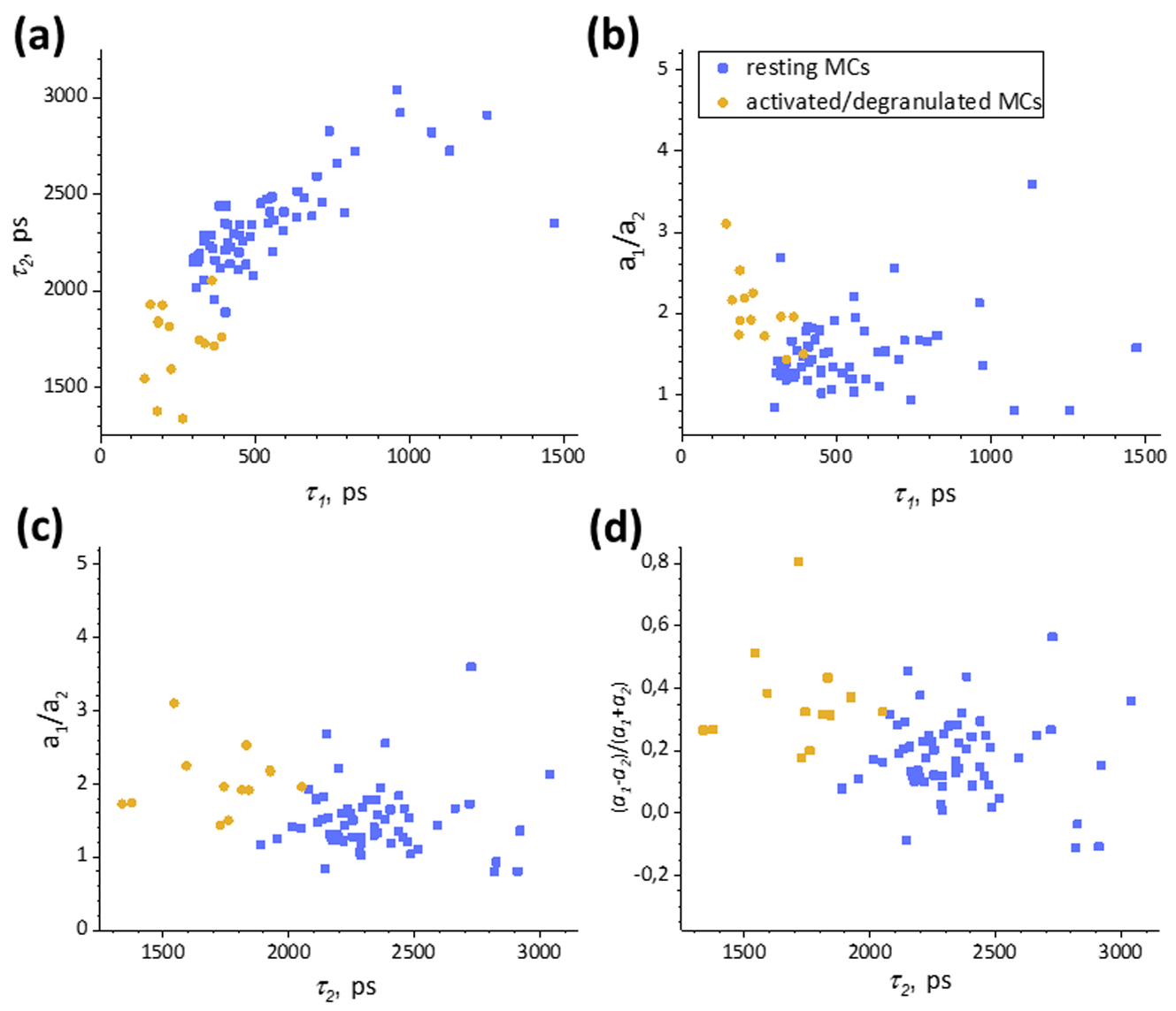


**Supplementary Fig. 2. *In vitro* segmentation of MC populations.** 2D segmentation of the *τ_1_*(*τ_2_*) (a), *τ_1_*(*a_1_*/*a_2_*) (b), *τ_2_*(*a_1_*/*a_2_*) (c) and *τ_2_*((*a_1_*-*a_2_*)/(*a_1_*+*a_2_*)) (d) TPE-FLIM parameters of the resting (blue squares) and activated/degranulated (orange circles) MCs measured *in vitro*.


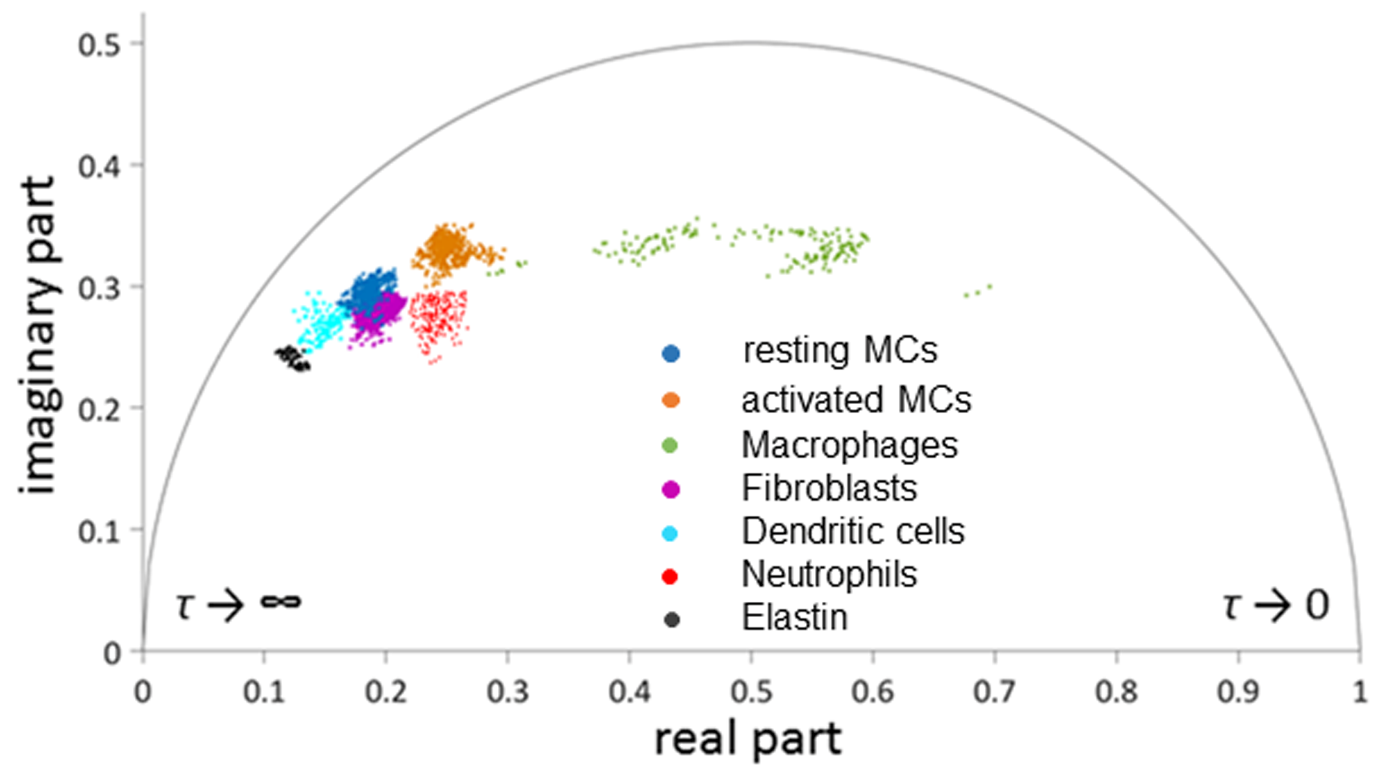


**Supplementary Fig. 3. Phasor plot of TPE-FLIM parameters of dermal components.** Phasor plot of exemplary resting MCs (blue points), activated MCs (orange points), macrophages (green points), fibroblasts (red points), neutrophils (red points) and dendritic cells (light blue points) measured *in vitro* in the culture. Elastin (grey points) was measured *in vivo* in the human dermis.


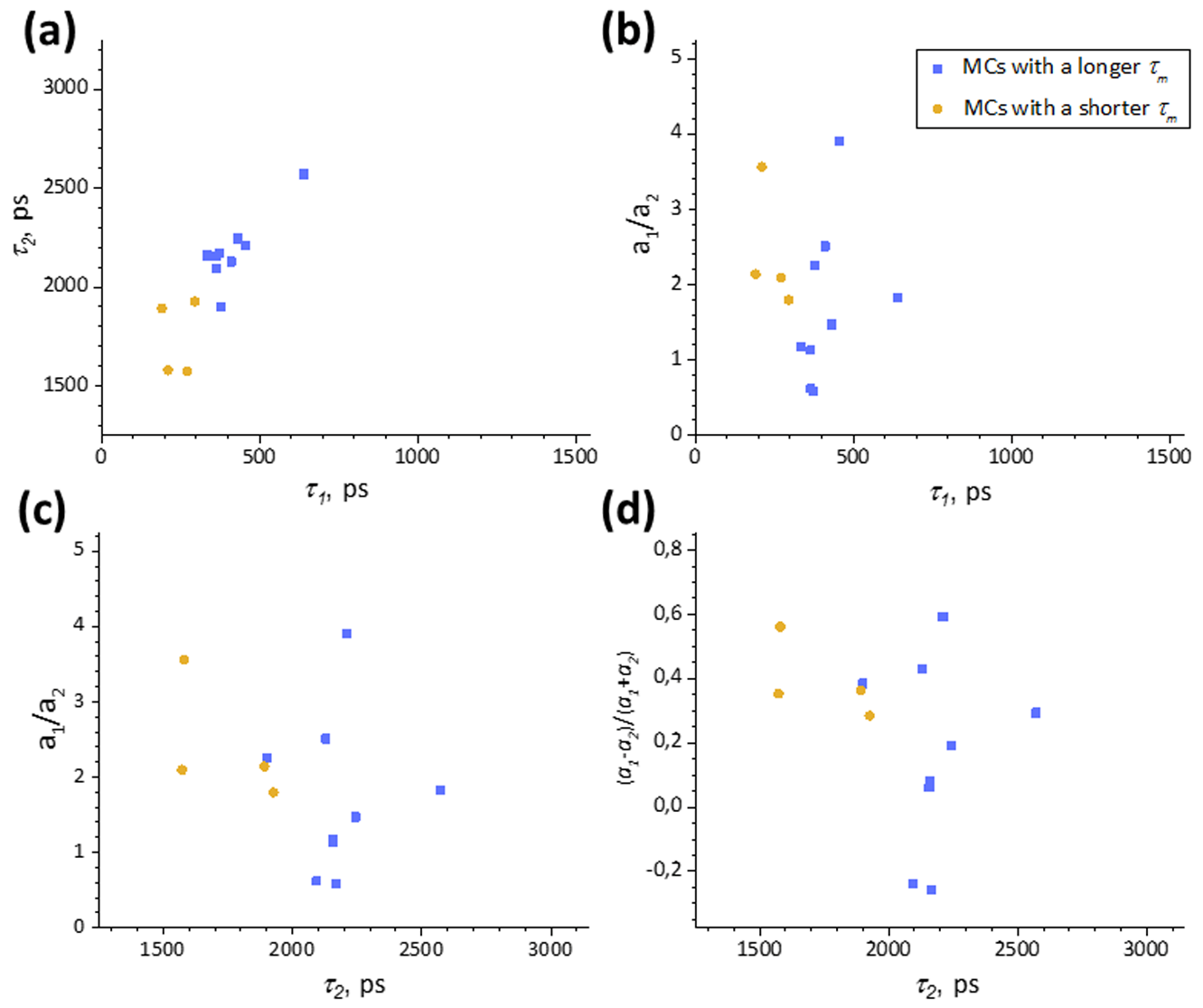


**Supplementary Fig. 4. *Ex vivo* segmentation of MC populations.** 2D segmentation of the *τ_1_*(*τ_2_*) (a), *τ_1_*(*a_1_*/*a_2_*) (b), *τ_2_*(*a_1_*/*a_2_*) (c) and *τ_2_*((*a_1_*-*a_2_*)/(*a_1_*+*a_2_*)) (d) TPE-FLIM signatures of the staining-proved MCs with a longer *τ_m_* (*n*=9, blue squares) and MCs with a shorter *τ_m_* (*n*=4, orange circles) measured *ex vivo* on the human skin cryo-sections.


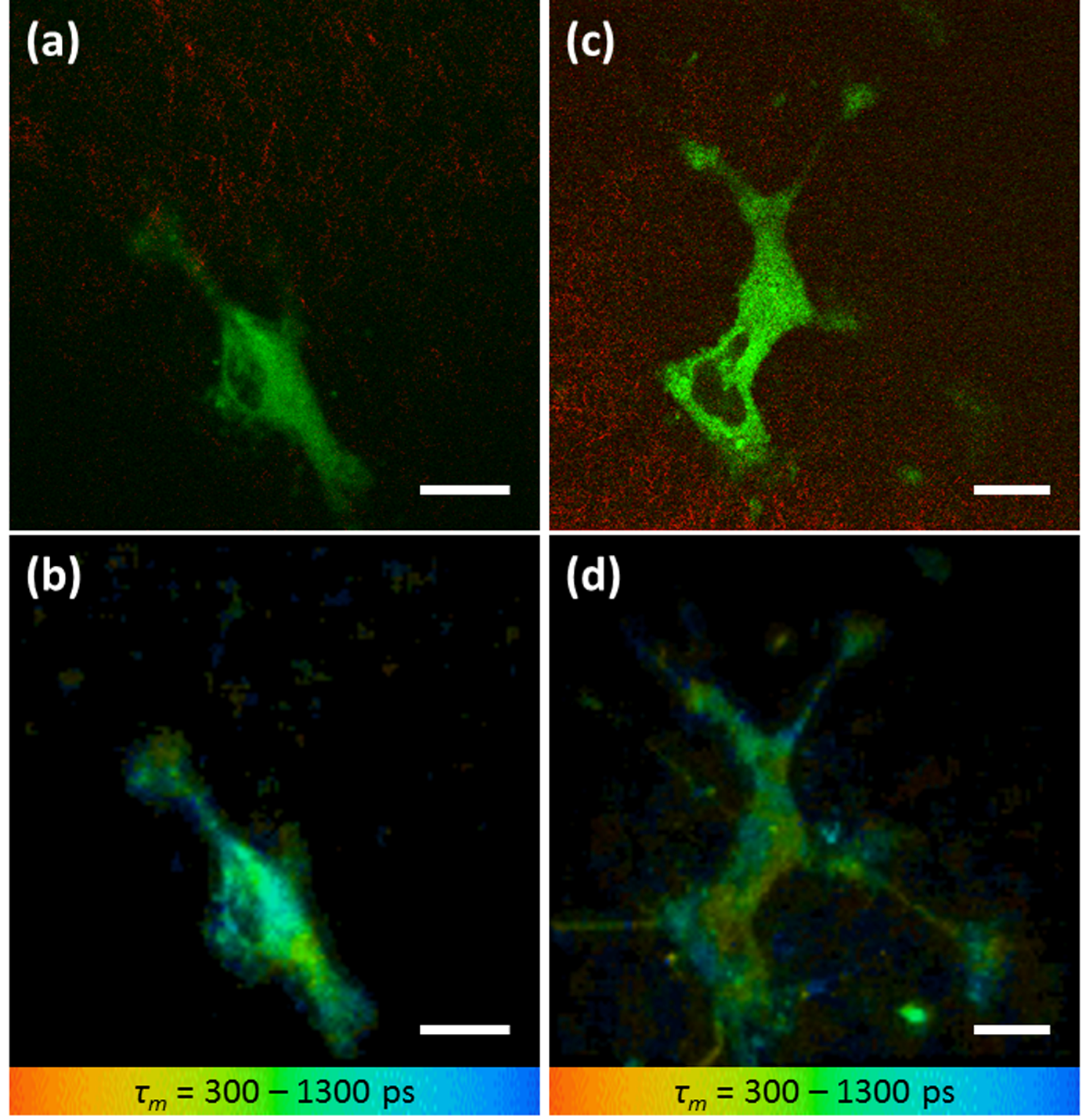


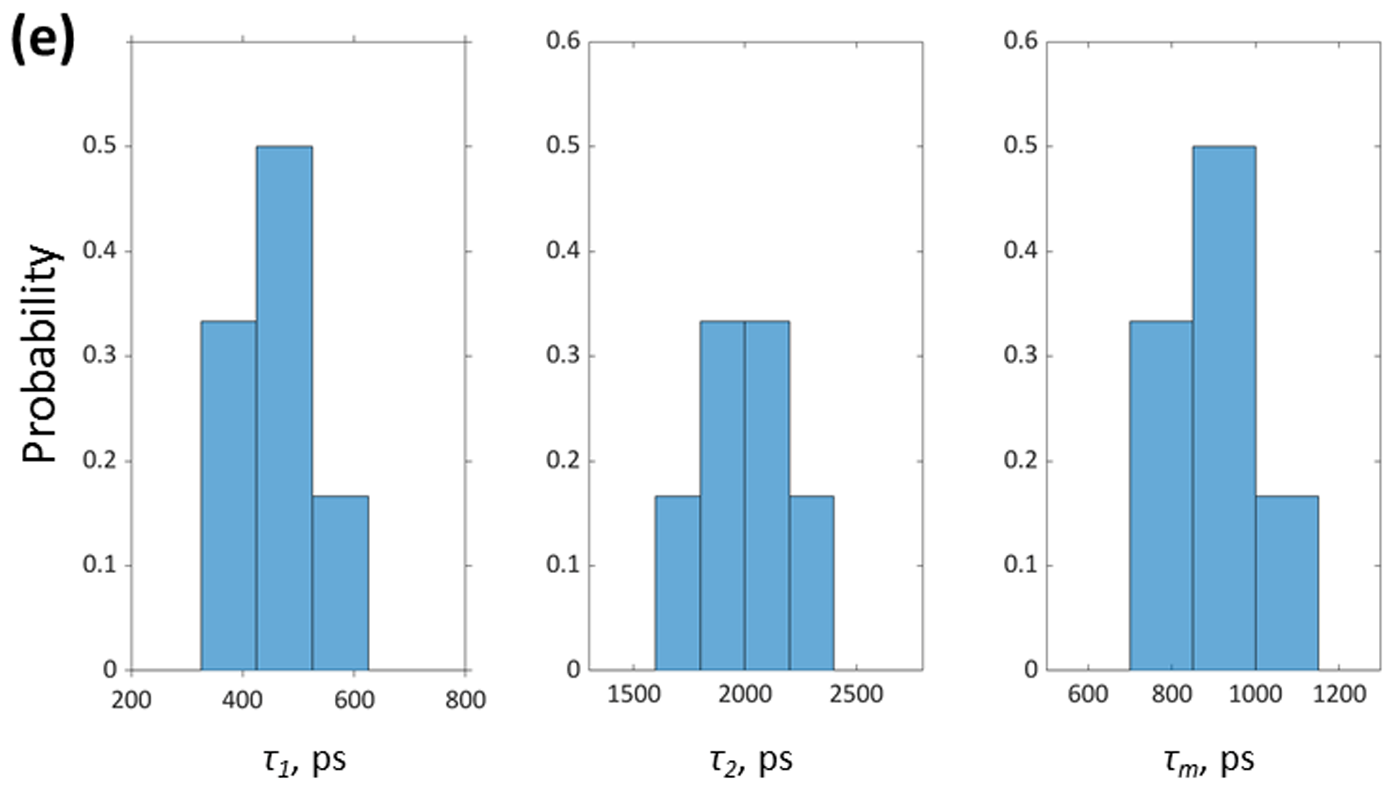


**Supplementary Fig. 5. TPE-FLIM imaging of fibroblasts.** Merged TPE-AF (green) and SHG (red) images of human fibroblasts in collagen solution measured *in vitro* (a, c) and corresponding TPE-FLIM images (b, d), showing mean fluorescence lifetime *τ_m_* in a color gradient from 300 to 1300 ps at 15 mW, 760 nm excitation. Image size is 58 µm (a, b) and 70 µm (c, d). (e) shows a histogram of TPE-FLIM parameters *τ_1_*, *τ_2_*, *τ_m_* measured for the six fibroblasts *in vitro*.


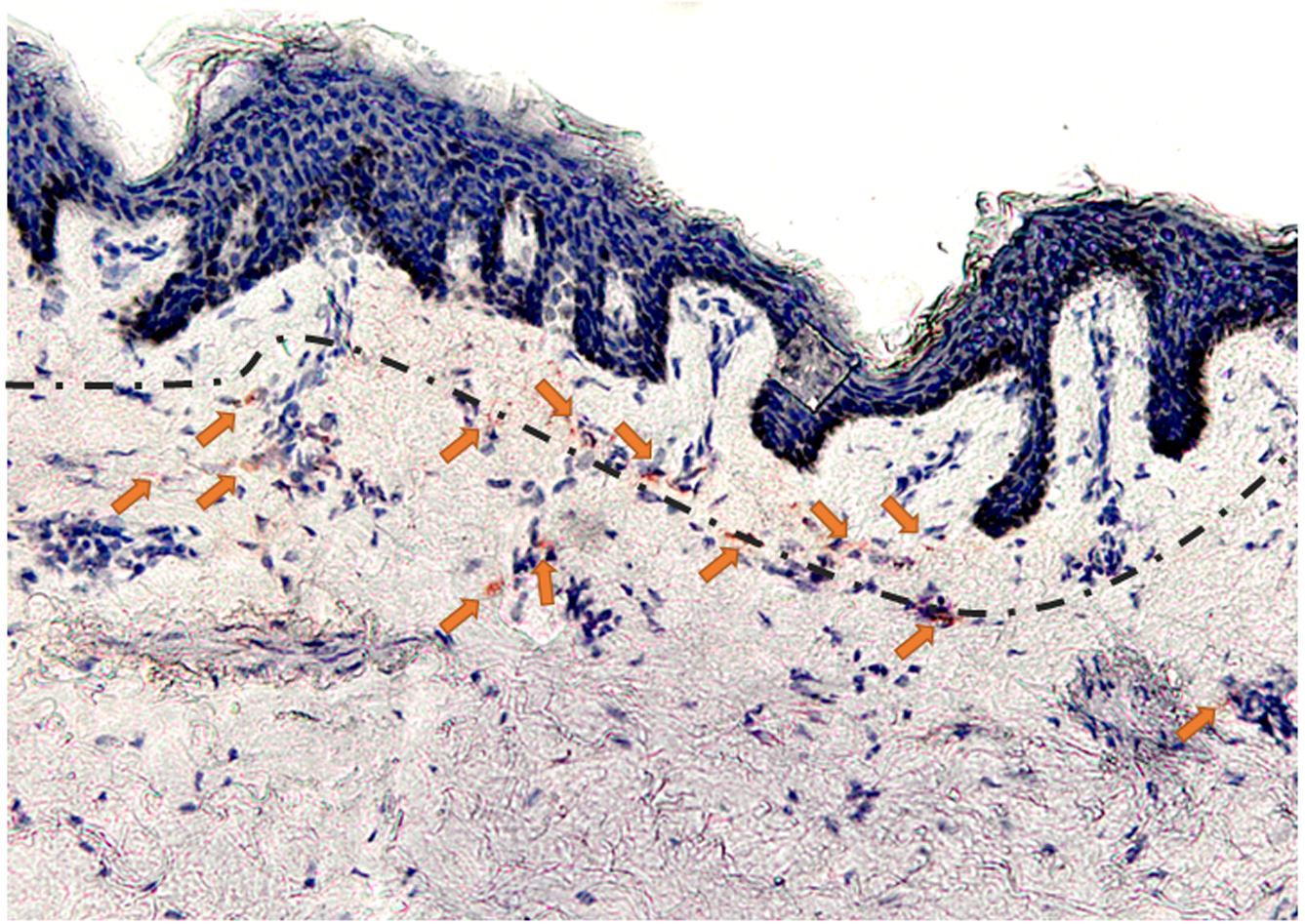


**Supplementary Fig. 6. MC stained cryo-section.** A typical brightfield microscopic image of a tryptase-stained skin biopsy cryo-section with counterstained nuclei Mayer's hemalum solution (Merck, Darmstadt, Germany). Stained MCs are labeled with the orange arrows. The border between the papillary and reticular dermis is marked with a black dashed line.


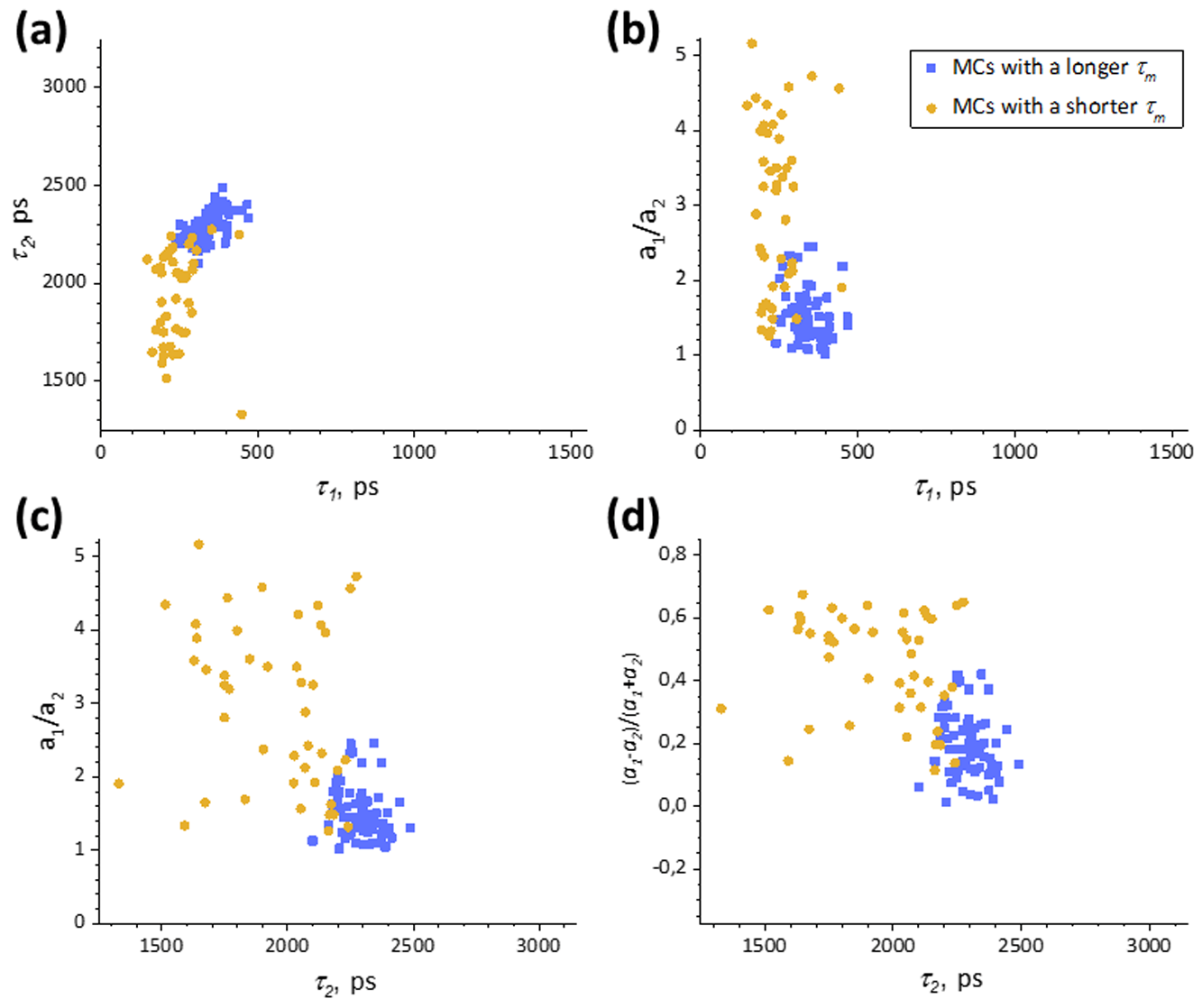


**Supplementary Fig. 7. *In vivo* segmentation of MC populations.** 2D segmentation of the *τ_1_*(*τ_2_*) (a), *τ_1_*(*a_1_*/*a_2_*) (b), *τ_2_*(*a_1_*/*a_2_*) (c) and *τ_2_*((*a_1_*-*a_2_*)/(*a_1_*+*a_2_*)) (d) TPE-FLIM parameters of the MCs with a longer *τ_m_* (*n*=71, blue squares) and MCs with a shorter *τ_m_* (*n*=48, orange circles) measured *in vivo* on the inner forearm of healthy volunteers.


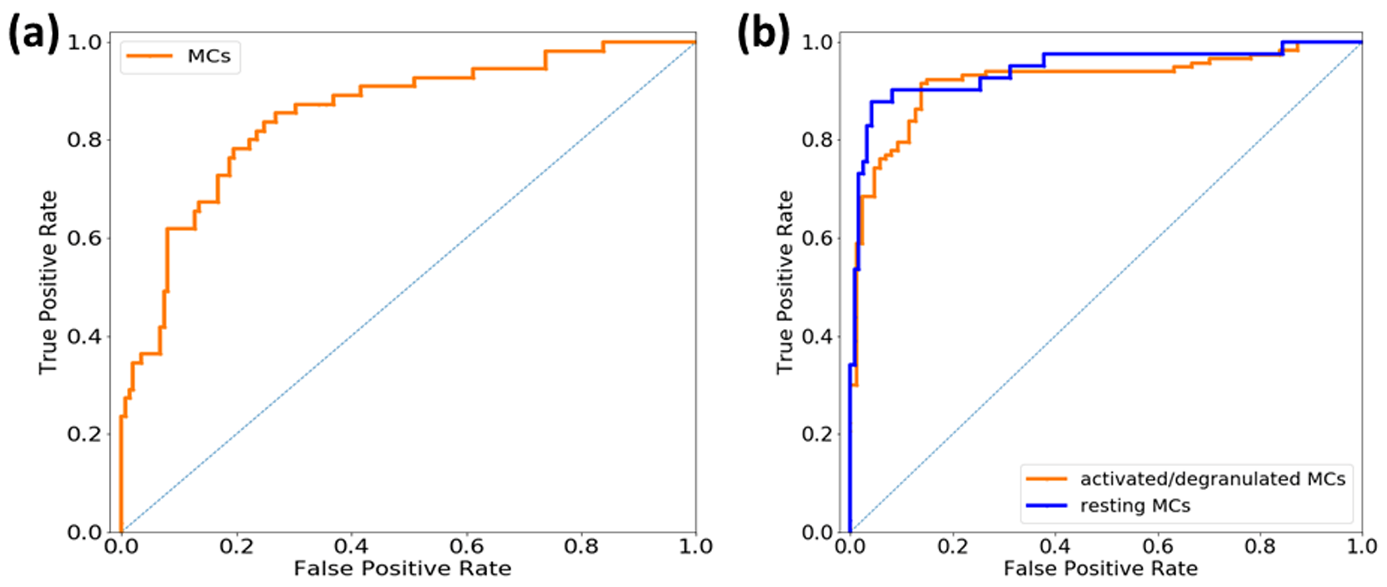


**Supplementary Fig. 8. ROC-curve for decision tree classifier for the classification of MCs.** The ROC-curve for the classification of MCs versus other dermal cells (a) and the ROC-curve for both resting and activated/degranulated MCs versus other dermal cells (b).
